# *Ustilago maydis* Serves as a Novel Production Host for the Synthesis of Plant and Fungal Sesquiterpenoids

**DOI:** 10.3389/fmicb.2020.01655

**Published:** 2020-07-24

**Authors:** Jungho Lee, Fabienne Hilgers, Anita Loeschke, Karl-Erich Jaeger, Michael Feldbrügge

**Affiliations:** ^1^Bioeconomy Science Centre, Cluster of Excellence on Plant Sciences, Institute for Microbiology, Heinrich Heine University Düsseldorf, Düsseldorf, Germany; ^2^Institute for Molecular Enzyme Technology, Heinrich Heine University Düsseldorf, and Forschungszentrum Jülich GmbH, Jülich, Germany; ^3^Institute of Bio- and Geosciences IBG-1, Biotechnology, Forschungszentrum Jülich GmbH, Jülich, Germany

**Keywords:** α-cuprenene, basidiomycete, lycopene, mevalonate pathway, (+)-valencene

## Abstract

Sesquiterpenoids are important secondary metabolites with various pharma- and nutraceutical properties. In particular, higher basidiomycetes possess a versatile biosynthetic repertoire for these bioactive compounds. To date, only a few microbial production systems for fungal sesquiterpenoids have been established. Here, we introduce *Ustilago maydis* as a novel production host. This model fungus is a close relative of higher basidiomycetes. It offers the advantage of metabolic compatibility and potential tolerance for substances toxic to other microorganisms. We successfully implemented a heterologous pathway to produce the carotenoid lycopene that served as a straightforward read-out for precursor pathway engineering. Overexpressing genes encoding enzymes of the mevalonate pathway resulted in increased lycopene levels. Verifying the subcellular localization of the relevant enzymes revealed that initial metabolic reactions might take place in peroxisomes: despite the absence of a canonical peroxisomal targeting sequence, acetyl-CoA C-acetyltransferase Aat1 localized to peroxisomes. By expressing the plant (+)-valencene synthase CnVS and the basidiomycete sesquiterpenoid synthase Cop6, we succeeded in producing (+)-valencene and α-cuprenene, respectively. Importantly, the fungal compound yielded about tenfold higher titers in comparison to the plant substance. This proof of principle demonstrates that *U. maydis* can serve as promising novel chassis for the production of terpenoids.

## Introduction

Terpenoids (isoprenoids) constitute an important class of secondary metabolites found mainly in plants and fungi. They are classified according to the number of C5 scaffold isopentenyl diphosphate (IPP) building blocks. Sesquiterpenoids, for example, contain three and diterpenoids consist of four of such building blocks, forming C15 and C20 scaffolds, respectively. Terpenoids exhibit a plethora of biological functions like photoprotection, hormone signaling and defense against pathogens (Schmidt-Dannert, 2015; Moser and Pichler, 2019; Troost et al., 2019). Because of these diverse bioactivities they are also of interest for biotechnology. Artemisinin, for example, functions as antimalarial drug and (–)-patchoulol serves as a valuable fragrance for the cosmetics industry. Lycopene and (+)-valencene are natural food additives. The latter also serves as precursor for the synthesis of the highly valuable aromatic substance nootkatone (Schempp et al., 2018; Moser and Pichler, 2019).

Traditionally, terpenoids are extracted from plant or fungal materials. Extensive efforts are made to produce these compounds heterologously for increased sustainability as well as to expand the chemical diversity of terpenoids by further modification of near-to-natural versions (Ro et al., 2006; Ignea et al., 2018; Schempp et al., 2018). Several companies like Amyris, Evolva, Isobionics (now BASF), and Firmenich have already marketed diverse terpenoids produced with engineered microorganisms (Schempp et al., 2018).

A rich source of terpenoids are higher basidiomycetes, forming mushrooms (Schmidt-Dannert, 2015; Xiao and Zhong, 2016). Prominent examples are derivatives of the sesquiterpenoid illudin S from *Omphalotus olearius* that exhibits strong anti-tumor activity (Jaspers et al., 2002), and the diterpenoid pleuromutilin from *Clitopilus passeckerianus* that has an antibacterial effect by inhibiting the large subunit of prokaryotic ribosomes (Hartley et al., 2009).

Most fungal terpenoids are not yet studied because it is difficult to obtain enough pure substances for defined biological assays. For the current access of the compounds, researchers rely mostly on improving the biosynthesis in the natural producer. However, higher basidiomycetes often entail the disadvantage of being difficult to cultivate and that sophisticated molecular tools for pathway engineering are not available. Alternatively, synthetic hosts like *Escherichia coli* or *Saccharomyces cerevisiae* are used (Xiao and Zhong, 2016). Despite their advantages in biotechnology, the production of antibacterial substances in *E. coli* is challenging and terpenoids from basidiomycetes might be toxic for ascomycetes like *S. cerevisiae*. Based on these reasons, we followed the strategy to exploit a well-studied basidiomycete for the production of terpenoids.

We chose the corn smut *Ustilago maydis* that serves as an excellent model system for basic cell biology and plant pathology (Kahmann and Kämper, 2004; Zarnack and Feldbrügge, 2010; Béthune et al., 2019). The genome is sequenced and well-annotated. Transcriptomics and proteomics have been carried out and sophisticated molecular tools are established (Kämper et al., 2006; Scherer et al., 2006; Koepke et al., 2011; Olgeiser et al., 2019). Strains can be efficiently generated by homologous recombination and after stable insertion in the genome, selection markers can be excised resulting in marker-free strains either to recycle the resistance marker or to obtain safe strains for biotechnological production (Khrunyk et al., 2010; Terfrüchte et al., 2014).

Besides serving as a model for basic research, *U. maydis* is also advancing as a flexible microorganism for biotechnological applications. Importantly, the yeast form is non-pathogenic. Furthermore, *U. maydis* only infects corn and infected ears have been eaten as a delicacy for centuries in Mexico, indicating that its consumption is not harmful to humans (Feldbrügge et al., 2013). The fungus constitutes a promising production chassis for a whole variety of biotechnologically relevant compounds including itaconic acid, a versatile building block for tailor-made biofuels (Wierckx et al., 2019; Becker et al., 2020). Additionally, it produces biosurfactants like mannosylerythritol lipids and ustilagic acid, which can be used as the basis for sustainable detergents and emulsifiers (Teichmann et al., 2007, 2010; Feldbrügge et al., 2013). A recent application is the establishment of *U. maydis* as a novel host for the production of heterologous proteins. This is strongly supported by the discovery that valuable proteins like antibody formats can be exported in the culture medium by a novel unconventional secretion pathway (Sarkari et al., 2014). This pathway prevents undesired N-glycosylation of the product that is usually processed during conventional secretion (Stock et al., 2012; Sarkari et al., 2014; Terfrüchte et al., 2018).

At present, *U. maydis* strains are being optimized to convert plant biomass into valuable products. Strains have been successfully engineered to grow on cellulose, xylose, and polygalacturonic acid (Geiser et al., 2016a; Stoffels et al., 2020). The latter is a major component of pectin. Thus, a consolidated bioprocess is being developed, in which complex natural substrates are converted to fermentable sugars and to value-added compounds in the future (Geiser et al., 2016a). Here, we add terpenoids to the growing list of compounds produced in *U. maydis*.

## Results

### *U. maydis* Contains an Evolutionarily Conserved FPP Pathway

**FIGURE 1:**
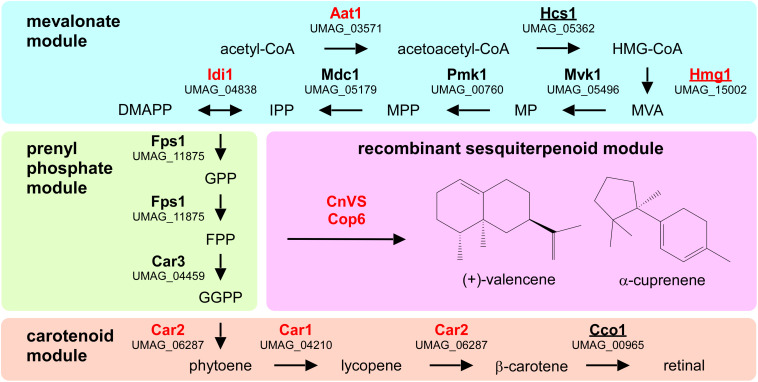
Metabolic network for the heterologous production of sesquiterpenoids. Graphical representation of the various modules involved in recombinant sesquiterpenoid synthesis: mevalonate module (blue), prenyl phosphate module (green), carotenoid module (orange), and recombinant sesquiterpenoid module (pink). Enzyme names are given in Table 1. Enzymes studied in this publication are indicated in red and those that were already studied in *U. maydis* are underlined (HMG-CoA, 3-hydroxy-3-methylglutaryl-CoA; MVA, mevalonate; MP, mevalonate-5-phosphate; MPP, mevalonate-pyrophosphate; IPP, isoprenyl-pyrophosphate; DMAPP, dimethylallyl-pyrophosphate, GPP, geranyl-pyrophosphate; FPP, farnesyl-pyrophosphate GGPP, geranylgeranyl-pyrophosphate).

To design a strategy for the heterologous production of sesquiterpenoids in *U. maydis*, we predicted the underlying metabolic pathways using bioinformatics analysis (Figure 1 and Supplementary Figure S1). As a starting point, we adopted information from the KEGG pathway “terpenoid backbone biosynthesis” for *U. maydis* (Kyoto Encyclopedia of Genes and Genomes^1^, Kanehisa and Goto, 2000). We conceptually divided the metabolic network into four parts: the mevalonate module, the prenyl phosphate module, the carotenoid module, and the recombinant sesquiterpenoid module (Troost et al., 2019). The mevalonate pathway from acetyl-CoA to isopentyl- and dimethylallyl diphosphate (IPP and DMAPP) is evolutionarily highly conserved in eukaryotes (Miziorko, 2011). Therefore, we used the detailed knowledge on *S. cerevisiae*, *H. sapiens*, and *A. thaliana* as a blueprint (Figure 1, Table 1, and Supplementary Figure S1; Nielsen and Keasling, 2016; Ye et al., 2016). The enzymatic functions of enzymes Hcs1 (3-hydroxy-3-methylglutaryl-CoA-synthase) and Hmg1 (3-hydroxy-3-methylglutaryl-coenzyme A reductase) have been studied previously in *U. maydis* (Croxen et al., 1994; Winterberg et al., 2010). The remaining enzymes (acetyl-CoA-C-acetyltransferase Aat1, mevalonate kinase Mvk1, phosphomevalonate kinase Pmk1, mevalonate diphosphate decarboxylase Mdc1, IPP isomerase Idi1, and farnesyl pyrophosphate synthase Fps1) were identified by high amino acid sequence similarity and the presence of conserved domains when compared to well-studied fungal, human and plant versions (Figure 1, Table 1, and Supplementary Figure S1). Mevalonate kinase was the only enzyme not predicted in the KEGG pathway database. The same strategy was applied for the identification of enzymes of the prenyl phosphate module, producing geranylgeranyl-diphosphate via chain elongation from IPP and DMAPP (GGPP; Figure 1 and Table 1).

**TABLE 1:** Putative or experimentally verified enzymes of the mevalonate pathway in different organisms.

*U. maydis*			*S. cerevisiae*			*H. sapiens*		
Name	UMAG	NCBI annotation	Name	Identities (%)	e-value	Name	Identities (%)	e-value
Aat1	03571	Acetyl-CoA C-acetyltransferase	Erg10p	209 (52)	2e-128	ACAT1	240 (59)	4e-166
						ACAT2	175 (45)	7e-104
Hcs1	05362	Probable hydroxymethylglutaryl-CoA synthase	Erg13p	219 (48)	1e-154	HMGCS1	234 (50)	3e-154
						HMGCS2	228 (49)	1e-147
Hmg1	15002	Probable 3-hydroxy-3-methylglutaryl-CoA reductase	Hmg1p	273 (62)	1e-179	HMGCR	258 (60)	8e-172
			Hmg2p	278 (60)	0			
Mvk1	05496	Mevalonate-5-kinase	Erg12p	147 (37)	1e-60	MVK	148 (38)	3e-53
Pmk1	00760	Phosphomevalonate kinase	Erg8p	150 (29)	2e-40	PMVK	No homologs found	
Mdc1	05179	Mevalonate-5-pyrophosphate decarboxylase	Mvd1p	198 (50)	2e-119	MVD	200 (49)	3e-112
Idi1	04838	Isopentenyl diphosphate isomerase	Idi1p	137 (52)	2e-77	IDI1	116 (53)	3e-68
						IDI2	98 (42)	2e-51
Fps1	11875	Putative bifunctional (2E,6E)-farnesyl diphosphate synthase/dimethylallyl transtransferase	Erg20p	211 (69)	9e-155	FDPS	169 (50)	8e-103

The carotenoid module has been predicted before and its main function is the production of retinal that serves as chromophore for photoactive opsin channels Ops1-3 (Estrada et al., 2009; Panzer et al., 2019). The cleavage reaction of β-carotene into two retinal molecules is catalyzed by Cco1 (β-carotene cleavage oxygenase; Estrada et al., 2009). It was already shown that deletion of *cco1* resulted in the accumulation of β-carotene (Estrada et al., 2009). Loss of retinal synthesis causes no mutant phenotype under standard growth conditions or during pathogenic development, thus the biological function of opsins in *U. maydis* is as yet unclear (Estrada et al., 2009; Panzer et al., 2019).

For the recombinant sesquiterpenoid module, we aimed to branch off the key precursor farnesyl pyrophosphate (FPP). We chose the plant (+)-valencene synthase from *Callitropsis nootkatensis* (CnVS; Troost et al., 2019) and the fungal α-cuprenene synthase Cop6 from *Coprinopsis cinerea* (Agger et al., 2009) to synthesize (+)-valencene and α-cuprenene, respectively (Figure 1). In essence, (+)-valencene served as a benchmarking product for our new approach as it has been the target in multiple studies on microbial sesquiterpenoid production before, while α-cuprenene served as an example of a basidiomycete compound that has been well-studied before (Agger et al., 2009; Beekwilder et al., 2014; Frohwitter et al., 2014; Stöckli et al., 2019; Troost et al., 2019).

### Establishing the Production of Lycopene in *U. maydis* as an Indicator of Carotenoid Precursors

**FIGURE 2:**
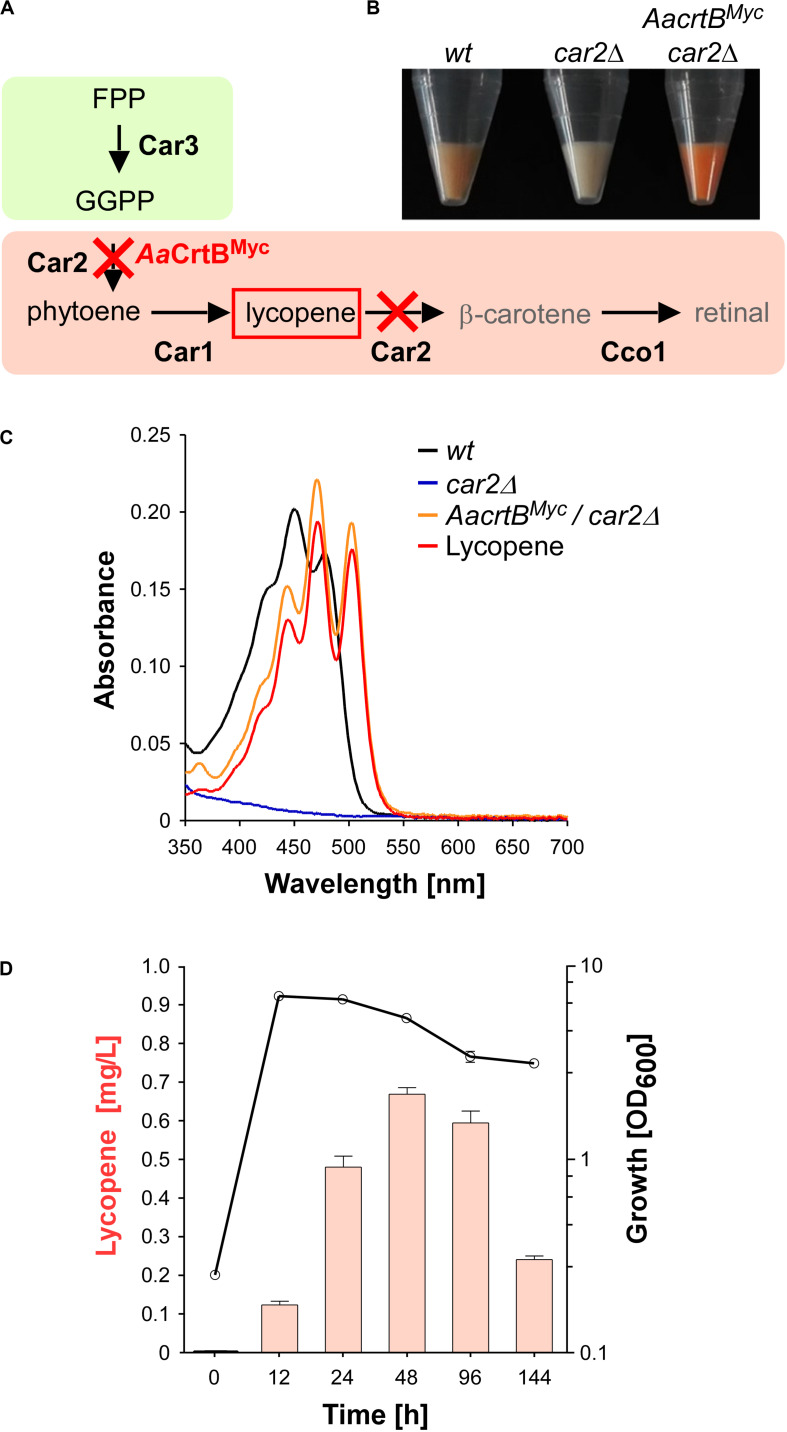
Lycopene production in *U. maydis*. **(A)** Schematic representation of the carotenoid module given in Figure 1 (red cross indicates gene deletion; *Aa*CrtB^Myc^ is the phytoene synthase from *Agrobacterium aurantiacum* containing a triple Myc epitope tag). **(B)** Cell pellets of strains indicated above the image. **(C)** Absorption spectrum of various *U. maydis* strains (genotype as indicated). **(D)** Analysis of lycopene concentrations (left, orange bars) in relation to the growth phase of strain producing *Aa*CrtB^Myc^ and carrying a deletion of *car2* (OD_600_, black line). Three independent biological experiments (*n* = 3) were carried out. Error bars indicate standard deviation of the mean (SD).

For the production of recombinant sesquiterpenoids, we aimed to increase the activities of the mevalonate module in order to obtain higher levels of the key precursor FPP (Figure 1). However, FPP is a toxic intermediate and the detection of intracellular FPP levels is not trivial (Dahl et al., 2013). Therefore, we decided to modify the intrinsic carotenoid module to accumulate lycopene, a colored FPP-derived product that can be easily detected and quantified (Figures 2A,B). Carotenoid synthesis should serve as an easy read-out system for the activity of the underlying metabolic pathway as well as a safety valve for high FPP levels.

As mentioned above, the carotenoid module was previously studied in *U. maydis* (Figures 1, 2A). Lycopene is naturally produced by desaturation of phytoene, and then further converted in two steps, i.e., cyclisation forming β-carotene and the cleavage of this intermediate to retinal. Car1 is the phytoene desaturase (Estrada et al., 2009) and deletion of *car1* resulted in loss of carotenoid accumulation (Supplementary Figures S2A,B). Notably, in fungi, Car2 is a bifunctional enzyme, serving as phytoene synthase and lycopene cyclase. In preparation of lycopene production, we deleted *car2* in the wild type, which also abolished carotenoid accumulation due to the bifunctionality of the encoded enzyme (Figures 2A–C and Supplementary Figures S2A,B). The resulting strains exhibited no growth defects. This is consistent with the observation that *cco1Δ* strains and opsins are dispensable for normal growth of *U. maydis* (Estrada et al., 2009). In order to synthesize lycopene in the *car2Δ* strain, we produced a heterologous phytoene synthase from *Agrobacterium aurantiacum* (*Aa*CrtB; Chen et al., 2016). This should enable heterologous reconstruction of the process and efficient visual detection of the red color of lycopene (Figures 2A,B). The corresponding bacterial open reading frame was codon-optimized for *U. maydis* (see the section “Materials and Methods”) and expression was controlled by the constitutively active promoter P*_*rpl40*_*. The respective promoter region was derived from *rpl40*, encoding a ribosomal protein of the large subunit. The resulting construct was inserted at the *car2* locus of the *car2Δ* strain by homologous recombination. In order to confirm production of the full length protein, a triple Myc epitope tag was fused at the C-terminus. The production was verified by Western blot analysis (Supplementary Figures S3A, S4A).

Analyzing the resulting strain demonstrated the production of lycopene (Figures 2B,C). Recording an absorption spectrum of cell extracts in *n*-hexane showed that the spectrum was shifted in comparison to the wild type from β-carotene- to lycopene-specific maxima (λ_max_ 450 nm and 503 nm, respectively; Fish et al., 2002), as measured with a commercially available lycopene standard (see the section “Materials and Methods”). Studying the production in shake flasks over time revealed that the lycopene titer increased during cell proliferation. Lycopene was still produced in the stationary phase and maximal amounts were detected after 48 h of cultivation (Figure 2D; 0.7 mg/L; see the section “Materials and Methods”). Production of *Aa*CrtB^Myc^ in a *car1* and *car2* double deletion strain resulted in no production of carotenoids and confirmed the necessity of Car1 for implementation of lycopene as end product in our strategy (Supplementary Figure S2B). In summary, heterologous production of a bacterial phytoene synthase in a genetically engineered strain resulted in the efficient production of lycopene as indirect molecular read-out for intracellular FPP levels.

### Metabolic Engineering of the Mevalonate Pathway Monitored by Lycopene Production

In order to increase the activity of the metabolic pathways leading to higher FPP levels, we altered the expression of three biosynthetic genes that were known to encode enzymes with limiting activity in other well-studied systems (Nielsen and Keasling, 2016; Ye et al., 2016): Aat1, Hmg1 and Idi1 (Figure 1 and Table 1). In the case of Hmg1, we generated an N-terminal truncated version of the reductase designated Hmg1^N^*^Δ^*^1–932^. This enzyme is known to be a rate-limiting enzyme in other organisms, whose expression is under tight control (DeBose-Boyd, 2008). It contains a targeting peptide in the N-terminal extension for insertion into the ER membrane and deletion of this region resulted in cytoplasmic localization and higher activity (Donald et al., 1997; Polakowski et al., 1998; Kampranis and Makris, 2012; truncation indicated in Supplementary Figure S1). In contrast to *S. cerevisiae*, *U. maydis* contains a single Hmg1 enzyme (Table 1) and its activity was already investigated in *E. coli* (Croxen et al., 1994).

**FIGURE 3:**
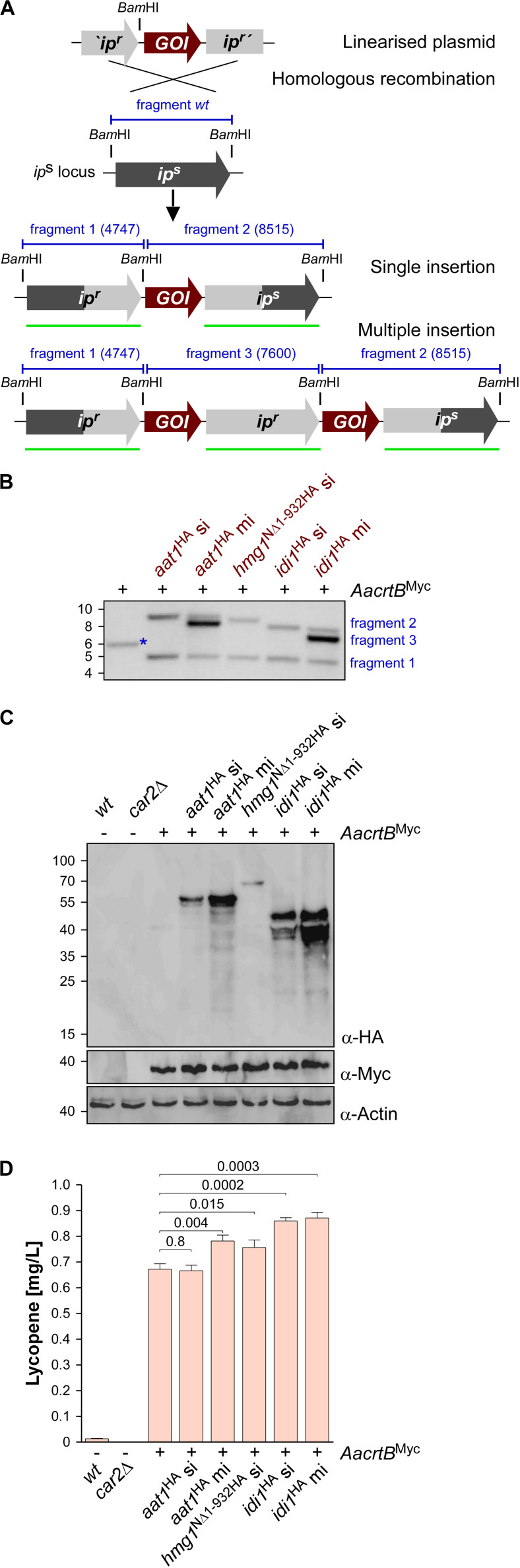
Genetic engineering of the mevalonate pathway. **(A)** Graphical representation of the genetic modification at the *ip*^*s*^ locus encoding an iron sulfur protein conferring carboxin resistance. The wild type (*wt*) version encoding a sensitive version *ip*^*s*^ is given in dark gray. The corresponding resistant version is given in light gray (*ip*^*r*^). The gene of interest (GOI) and fragments expected in Southern blot analysis (shown in **B**) are given in red and blue, respectively. The probe used for hybridization is indicated as a green.

To generate *U. maydis* strains with different levels of gene expression, we chose the *ip*^*s*^ locus (Loubradou et al., 2001). Expression was controlled by the strong constitutively active promoter P*_*otef*_* and the respective open reading frames were fused at their N-terminus with a triple HA epitope tag for detection (Brachmann et al., 2004). For each selected candidate, namely *aat1*, *hmg1*, and *idi1*, we individually transformed linearized plasmids to generate three independent strains. We expected two types of homologous recombination events: (i) single or (ii) multiple insertion (Figure 3A). The type of homologous insertion was verified by Southern blot analysis (Figure 3B). The detection of the insertion event was designed in such a way that in case of a single insertion of the gene-of-interest the wild type fragment was replaced by two fragments of the predicted size. In case of a multiple insertion a third fragment of intermediary size is detected (Figures 3A,B and Supplementary Figure S2C). In order to verify protein amounts, we performed Western blot analysis and, as expected, multiple insertion resulted in higher protein amounts (Figure 3C). In the case of Hmg1^N^*^Δ^*^1–932HA^, we obtained only single insertion events, although thirty transformants were screened. This suggests that a strong production of a truncated Hmg1 interferes with growth.

Assaying the lycopene concentrations after 48 h of incubation in shake flasks revealed that in all cases of additional production of Aat1^HA^, Hmg1^N^*^Δ^*^1–932HA^, and Idi1^HA^ a statistically significant increase (Figure 3D). In the case of Aat1^HA^ producing strains, multiple insertion led to a higher lycopene production than a single insertion, indicating that mRNA and protein amounts were limiting. In the case of Hmg1^N^*^Δ^*^1–932HA^ we observed a slight increase in lycopene yield (Figure 3D). A titer of up to 0.9 mg/L could be achieved in strains producing Idi1^HA^. The amount of lycopene in the strain with multiple insertion of *idi1*^HA^ was not higher, indicating that most likely the enzyme activity, not the protein amount, is limiting or that this was not the limiting step in lycopene synthesis of this strain (Figure 3D, see the section “Discussion”). Thus, by addressing known bottlenecks of the mevalonate pathway, we were able to alter terpenoid production, which was easily measured as lycopene production. Hence, lycopene is a good indicator and an efficient as well as robust read-out system for tuning the precursor biosynthetic pathway.

### The Subcellular Localization of Enzymes Involved in FPP Synthesis

**FIGURE 4:**
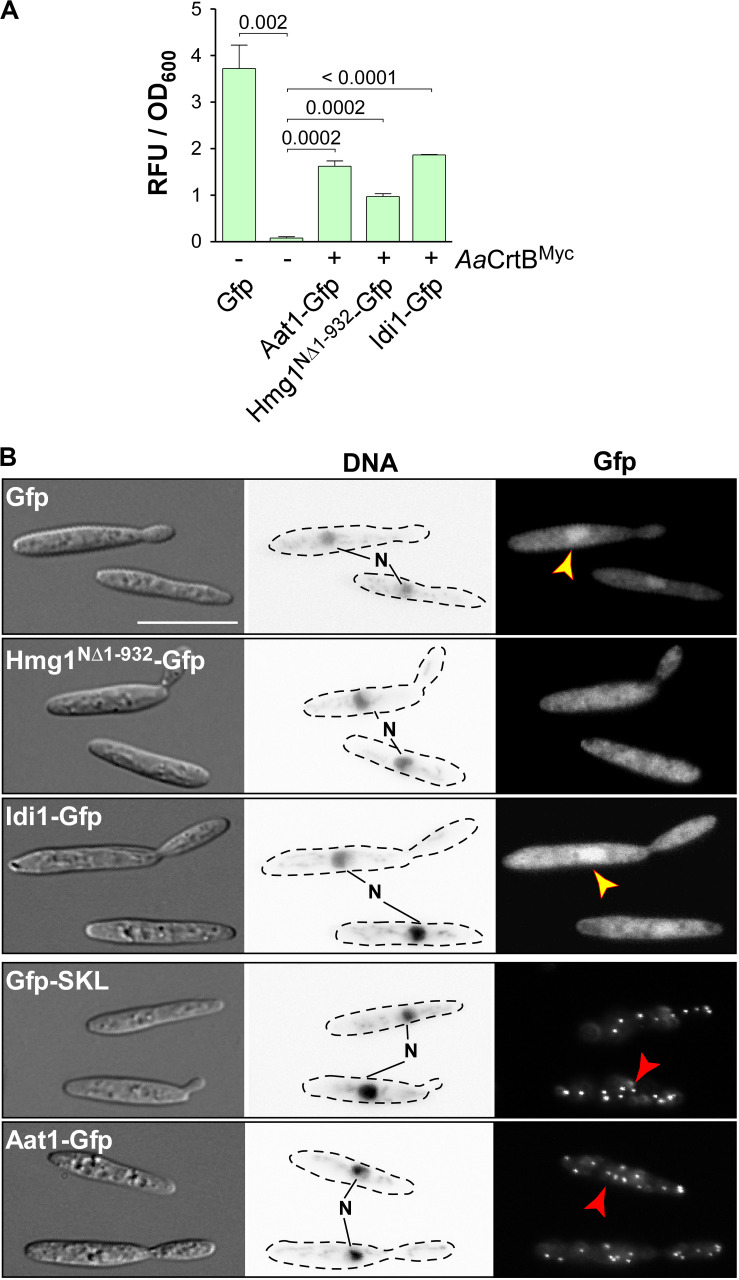
Subcellular localization of enzymes of the mevalonate pathway. **(A)** Quantification of Gfp production using fluorimeter measurements. Relative fluorescence units are given relative to the optical density (OD_600_). At least three independent biological experiments (*n* = 3) were performed with three technical replicates per strain. Error bars indicate standard error of the mean (SEM). Statistical significance was calculated using the unpaired two-tailed *t*-test and *p-*values were indicated above. Note, that the *Aa*CrtB^Myc^ producing strains carried a deletion of *car2*. **(B)** Microscopic analysis showing DIC images of fixed cells on the left (size bar, 10 μm). Corresponding staining of DNA with Hoechst 33342 (middle panel; N, nucleus; inverted image) and green fluorescence (Gfp) on the right (yellow and red arrowheads indicate nuclei and peroxisomes, respectively).

In other organisms, it has been reported that the mevalonate pathway is compartmentalized. In plants, for example, Aat1 homologs are found in peroxisomes as well as the cytoplasm (Wang et al., 2017). To study the subcellular localization of the respective enzymes in *U. maydis*, we fused Gfp at their N-termini (enhanced version of the green fluorescent protein, Clontech). We inserted the respective genes at the *ip*^*s*^ locus and used the constitutively active promoter P*_*tef*_* for expression (see Supplementary Table S1). Measuring green fluorescence revealed that the production of Gfp fusion proteins was detectable (Figure 4A). In Western blot analysis, we verified the expected protein size of the full-length fusion proteins (Supplementary Figure S2D).

Fluorescence microscopy showed that the truncated version Hmg1^N^*^Δ^*^1–932^-Gfp, missing the predicted ER membrane spanning region, localized mainly in the cytoplasm, like unfused Gfp. Idi1-Gfp also exhibited mainly cytoplasmic localization (Figure 4B). Thus, in contrast to plants (Simkin et al., 2011), geranyl pyrophosphate (GPP) appears to be synthesized in the cytoplasm. Idi1-Gfp also localized to a certain extent to the nucleus (Figure 4B). The same holds true for Gfp and it is known that a small proportion of Gfp and small Gfp fusion proteins mislocalize to the nucleus in *U. maydis* (Figure 4B, see below).

Microscopic observation of Aat1-Gfp revealed the accumulation of fluorescence signals in distinct foci (Figure 4B). This localization pattern is specific for peroxisomes, as indicated by the localization of Gfp carrying a C-terminal SKL localization signal or of peroxisomal protein Pex3-Gfp (Supplementary Figure S2E). A second characteristic for peroxisomes in *U. maydis* is their microtubule-dependent transport during hyphal growth (Supplementary Figure S2F; Guimaraes et al., 2015). Fluorescence signals of Aat1-Gfp moved processesively along the hypha as it is known for peroxisomes (comparison with movement of Gfp-SKL shown in Supplementary Figure S2F). Thus, either the initial reaction of the mevalonate pathway takes place in peroxisomes or an alternative enzyme is acting during FPP synthesis in the cytoplasm (see the section “Discussion”). In essence, studying the subcellular localization of enzymes of the mevalonate module is highly informative to devise future strategies for pathway engineering.

### Recombinant Production of the Plant Sesquiterpenoid (+)-Valencene in *U. maydis*

**FIGURE 5:**
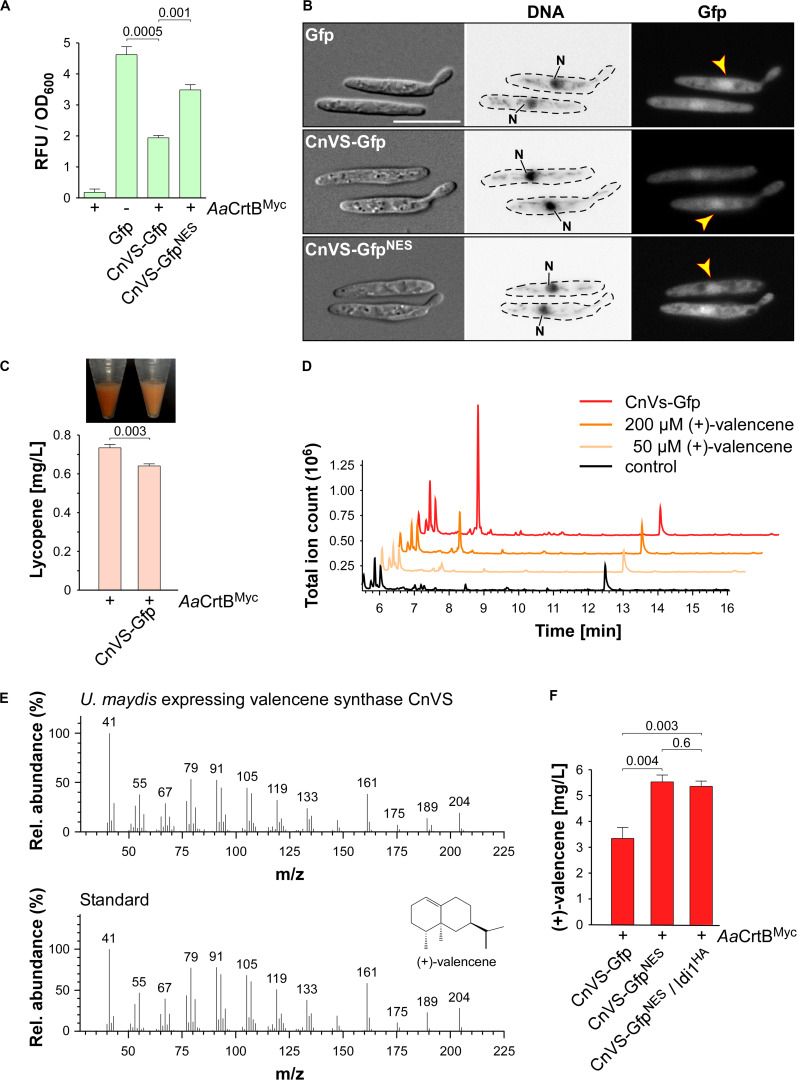
(+)-Valencene synthesis in *U. maydis*. **(A)** Quantification of Gfp production using fluorimeter measurements. Relative fluorescence units are given relative to the optical density (OD_600_). At least three independent biological experiments (*n* = 3) were performed with three technical replicates per strain. Error bars indicate standard error of the mean (SEM). Statistical significance was calculated using the unpaired two-tailed *t*-test and *p-*values were indicated above. Note, that the *Aa*CrtB^Myc^ producing strains carried a deletion of *car2*. **(B)** Microscopic analysis showing DIC images of fixed cells on the left (size bar, 10 μm). Corresponding staining of nuclear DNA with Hoechst 33342 (middle panel; N, nucleus; inverted image) and green fluorescence (Gfp) on the right (yellow arrowheads indicate nuclei). **(C)** Cell pellets and lycopene concentrations (orange bars) of strains given at the bottom. Three independent biological experiments (*n* = 3) were carried out. Error bars indicate standard deviation of the mean (SD). Statistical significance was calculated using the unpaired two-tailed *t*-test and *p-*values were indicated above. Note, that the *Aa*CrtB^Myc^ producing strains carried a deletion of *car2*. **(D)** GC-MS chromatogram of (+)-valencene from CnVS producing strain and the corresponding standard diluted in *n*-dodecane samples of the negative control. **(E)** Fragmentation pattern of peaks at 7.3 min (shown in **D**). **(F)** Concentration of (+)-valencene produced in the culture, determined by GC-FID according to commercial reference compound (Supplementary Figure S3B). Three independent biological experiments (*n* = 3) were carried out. Error bars indicate standard deviation of the mean (SD). Statistical significance was calculated using the unpaired two-tailed *t*-test and *p-*values were indicated above. Note, that the *Aa*CrtB^Myc^ producing strains carried a deletion of *car2*.

As proof of principle for sesquiterpenoid production in *U. maydis*, we chose to produce (+)-valencene via heterologous production of the plant (+)-valencene synthase CnVS, converting FPP to (+)-valencene (Figure 1; Troost et al., 2019). For efficient detection of protein production, we fused CnVS with Gfp at the N-terminus, as C-terminal fusions were reported to affect enzyme activity (Kampranis and Makris, 2012). We used the constitutively active promoter P*_*otef*_* and inserted the gene at the *upp3* locus of a strain producing *Aa*CrtB^Myc^ and carrying a deletion of *car2* (see Supplementary Table S1). Production was verified by fluorimeter measurements and Western blot analysis (Figure 5A and Supplementary Figure S3A).

Studying the subcellular localization showed the presence of CnVS-Gfp in the cytoplasm in order to ensure substrate access, but also to some extend in the nucleus (Figure 5B). To enhance cytoplasmic localization, we fused a nuclear export signal (NES) from murine minute virus (MTKKFGTLTI; Engelsma et al., 2008) to the N-terminus of Gfp resulting in CnVS-Gfp^NES^. Fluorescence microscopy revealed that the protein was also produced in this form but its nuclear localization was only slightly reduced (Figure 5B). Hence, the heterologous NES did not function efficiently in *U. maydis*. However, we observed that the protein amount of CnVS-Gfp^NES^ was significantly higher than CnVS-Gfp (Figure 5A and Supplementary Figure S3A). The N-terminal NES sequence most likely improved production or protein stability as a side effect.

Measuring the lycopene titer of the strain co-producing CnVS-Gfp and *Aa*CrtB^Myc^ (*car2Δ* background) showed a significant decrease of lycopene compared to the progenitor strain, which can also be detected by visual inspection (Figure 5C). Apparently, a proportion of the FPP is no longer available for lycopene synthesis, suggesting the functionality of the heterologous CnVS-Gfp. In order to measure the volatile (+)-valencene, we incubated strains with *n*-dodecane to collect the product (see the section “Materials and Methods”). After 48 h of incubation in shake flasks, we measured (+)-valencene in the *n*-dodecane phase by gas chromatography (Figure 5). We observed a prominent peak in GC-MS analysis with identical retention time of 7.3 min to the commercial reference (+)-valencene. Importantly, this peak was absent in the negative control strain (Figure 5D). Furthermore, analyzing a fragmentation pattern in mass spectrometry (MS) identified a pattern of peaks characteristic for (+)-valencene (Figure 5E; Troost et al., 2019). Thus, the identified substance is most likely the desired product. Using a commercial reference (Merck), we generated a standard calibration curve to quantify the titer via GC-FID analysis (Supplementary Figure S3B). Up to 5.5 mg/L (+)-valencene could be obtained from the CnVS-Gfp^NES^ producing strain (*Aa*CrtB^Myc^/*car2Δ*; Figure 5F).

In order to verify whether the N-terminal fusion of Gfp interferes drastically with the enzyme activity, we compared the (+)-valencene titer from a CnVS-Gfp producing strain (3.3 mg/L) with that of a strain producing an untagged version (4.8 mg/L; Supplementary Figure S3C). Hence, the Gfp fusion only slightly reduced the enzyme activity. However, it would be advisable to use an untagged CnVS for an improved production strain.

Finally, we tested whether overproduction of Idi1^HA^, which improved the carbon flux within the mevalonate pathway (see above), can improve the titer of (+)-valencene. To this end, the corresponding gene was inserted at the *cco1* locus of the strain producing CnVS-Gfp^NES^. For *idi1*^HA^ expression, we used the constitutively active promoter P*_*rpl10*_*. The promoter region was derived from *rpl10*, encoding ribosomal protein 10 of the large subunit. The lycopene titer was increased to 0.9 mg/L (Supplementary Figure S3E), which is comparable to the values obtained when Idi1^HA^ was expressed at the *ip*^s^ locus (Figure 3D). However, (+)-valencene production was not increased in this strain (Figure 5F). For future attempts, it might be advantageous to downregulate production of *Aa*CrtB^Myc^ or Car3 to redirect more FPP to sesquiterpenoids (see the section “Discussion”). In essence, we succeeded in the production of the widely produced plant sesquiterpenoid (+)-valencene, which was chosen here as a common model compound, by reengineering the intrinsic FPP pathway.

### Recombinant Production of the Basidiomycete Sesquiterpenoid α-Cuprenene in *U. maydis*

**FIGURE 6:**
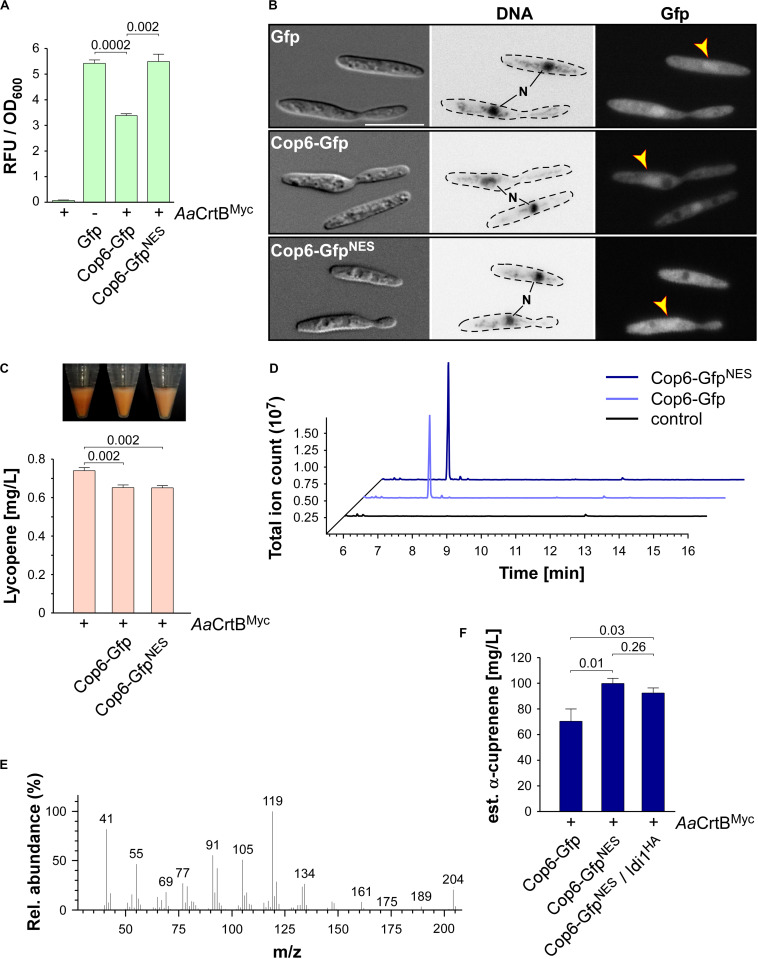
α-Cuprenene synthesis in *U. maydis*. **(A)** Quantification of Gfp production using fluorimeter measurements. Relative fluorescence units (RFU) are given relative to the optical density (OD_600_). At least three independent biological experiments (*n* = 3) were performed with three technical replicates per strain. Error bars indicate standard error of the mean (SEM). Statistical significance was calculated using the unpaired two-tailed *t*-test and *p-*values were indicated above. Note, that the *Aa*CrtB^Myc^ producing strains carried a deletion of *car2*. **(B)** Microscopic analysis showing DIC images of fixed cells on the left (size bar, 10 μm). Corresponding staining of DNA Hoechst 33342 (middle panel; N, nucleus, inverted image) and green fluorescence (Gfp) on the right (yellow arrowheads indicate nuclei). **(C)** Cell pellets and lycopene concentrations (orange bars) of strains given at the bottom. Three independent biological experiments (*n* = 3) were carried out. Error bars indicate standard deviation of the mean (SD). Statistical significance was calculated using the unpaired two-tailed *t*-test and *p-*values were indicated above. Note, that the *Aa*CrtB^Myc^ producing strains carried a deletion of *car2*. **(D)** GC-MS chromatogram of α-cuprenene from Cop6 producing strains. **(E)** Fragmentation pattern of peaks at 7.4 min given in **(D)**. **(F)** Estimated concentration of α-cuprenene determined via GC-FID using the reference compound β-caryophyllene (Supplementary Figure S4B). Three independent biological experiments (*n* = 3) were carried out. Error bars indicate standard deviation of the mean (SD). Statistical significance was calculated using the unpaired two-tailed *t*-test and *p-*values were indicated above. Note, that the *Aa*CrtB^Myc^ producing strains carried a deletion of *car2*.

As pointed out above, the basidiomycete *U. maydis* might serve as a suitable production platform for the synthesis of sesquiterpenoids from higher basidiomycetes. For this purpose, we chose to produce the sesquiterpenoid synthase Cop6 from the mushroom *C. cinerea* in *U. maydis*. The enzyme converts FPP into α-cuprenene (Agger et al., 2009; Figure 1). We fused Gfp or Gfp^NES^ to the N-terminus of Cop6, used the constitutively active promoter P*_*otef*_*, and targeted the corresponding construct to the *upp3* locus of a strain producing *Aa*CrtB^Myc^ and carrying a deletion of *car2* (see Supplementary Table S1). The production was confirmed via fluorimeter measurements and Western blot analysis (Figure 6A and Supplementary Figure S4A). Analysis of the subcellular localization revealed that Cop6-Gfp localized to the cytoplasm (Figure 6B). Consistent with the aforementioned localization of CnVS-Gfp^NES^, the NES version did not prevent nuclear accumulation but resulted in higher protein amounts (Figures 6A,B and Supplementary Figure S4A).

As was the case with production of CnVS described above, we observed that the amount of lycopene was reduced in strains co-producing the sesquiterpenoid synthase and *Aa*CrtB^Myc^ (*car2Δ* background), suggesting that part of the FPP was redirected to the recombinant sesquiterpenoid (Figure 6C). Respective strains were incubated for 48 h in shake flasks and the α-cuprenene was trapped in *n*-dodecane (see the section “Materials and Methods”). The GC-MS analysis of the organic phase showed an additional peak at a retention time of 7.4 min that was absent in the negative control (Figure 6D). In order to confirm α-cuprenene production, we analyzed the fragmentation pattern of this peak in mass spectrometry (Figure 6E). The fragmentation pattern was consistent with reported data for α-cuprenene (Agger et al., 2009). The total ion count was slightly higher in the NES version, most likely due to the higher enzyme amount in the Cop6-Gfp^NES^ producing strain (Figures 6A,B).

Finally, we estimated the yield of α-cuprenene production. Due to the absence of a commercial reference for α-cuprenene, we generated a standard calibration curve with the similar reference sesquiterpenoid β-caryophyllene (Merck) and compared the intensity of the electrically charged particles via GC-FID (Supplementary Figure S4B). We observed the highest amount of α-cuprenene in the Cop6-Gfp^NES^ producing strain and estimated a titer of 0.1 g/L (Figure 6F). Overproduction of Idi1^HA^ at the *cco1* locus did not increase the titer further (Figure 6F; see the section “Discussion”). In summary, we also succeeded in synthesizing the fungal sesquiterpenoid α-cuprenene, in addition to the plant-derived (+)-valencene, demonstrating that *U. maydis* serves as a promising novel host for the production of such specific sesquiterpenoids.

## Discussion

In this study, we present a straightforward strategy to engineer the terpenoid metabolism and produce the carotenoid lycopene as well as plant and fungal sesquiterpenoids in *U. maydis*. We have established lycopene production as an efficient read-out for the activity of the FPP-dependent pathway. This allowed initial pathway engineering and the production of plant (+)-valencene and fungal α-cuprenene as proof of principle.

### Establishing Lycopene Production as Molecular Read-Out, Reflecting Internal FPP Levels

To establish a terpenoid producing strain, we addressed early on the establishment of a simple read-out system for the activity of the FPP-based metabolic network. To this end, we redirected the intrinsic carotenoid pathway toward the production of lycopene by deletion of *car2* and heterologous production of the phytoene synthase *Aa*CrtB (Chen et al., 2016). Importantly, loss of retinal biosynthesis does not affect the growth of cells and currently, no light-regulated biological function could be assigned to retinal-dependent opsins (Estrada et al., 2009). Our strategy enabled visual and quantitative detection of the carotenoid lycopene, which was used for initial pathway engineering (see below). Genetic engineering that enhanced FPP synthesis, like overproduction of Idi1, resulted in increased lycopene levels. Conversely, production of sesquiterpenoid synthases consuming FPP reduced lycopene amounts. Thus, the intracellular FPP levels correlated with lycopene yields.

Similar strategies have been followed in *S. cerevisiae*, where GGPP was used as a metabolic branching point to synthesize carotenoids. The resulting strains were successfully applied to improve terpenoid production by carotenoid-based screening of mutants or by automated lab evolution (Ozaydin et al., 2013; Reyes et al., 2014; Trikka et al., 2015). Redirecting naturally occurring carotenoid pathways toward sesquiterpenoids was successfully achieved in *Corynebacterium glutamicum* and the red yeast *Xanthophyllomyces dendrorhous*. Both microorganisms are, in contrast to *S. cerevisiae*, natural producers of carotenoids (Melillo et al., 2013; Frohwitter et al., 2014).

In order to advance the system in the future, controlled down-regulation of GGPP synthase Car3 production will reduce its activity and channel higher amounts of FPP to terpenoid production. Regulated expression is advantageous since high FPP concentrations are toxic for microorganisms and increased pathway activity resulting in higher FPP levels could even limit production (Dahl et al., 2013). Thus, lycopene production serves as a safety valve for excess FPP. In *E. coli* this problem was addressed by using stress responsive promoters that respond to high FPP levels. The identified promoters were used to create a functional FPP biosensor and improved the production of amorphadiene (Dahl et al., 2013).

Finally, besides the use of lycopene as a read-out system, *U. maydis* might offer the possibility to serve as an alternative host for lycopene production. Such a sustainable biotechnological approach prevents the use of nutrient-rich food like tomatoes for lycopene extraction and avoids the risk of contamination by bacterial toxins when produced, for example, in *E. coli*. The lycopene production titer of our current system is rather low. However, pathway engineering in other fungal microorganisms like *S. cerevisiae* resulted in strains producing 0.3 g/L of lycopene in shake flask fermentation (Shi et al., 2019). Thus, comparable pathway engineering in *U. maydis* could result in similar increases of the yield.

### Genetic Engineering for Higher Biosynthesis of FPP

Initially, we carried out a bioinformatics analysis allowing us to annotate the mevalonate, prenyl phosphate, and carotenoid modules. As pointed out above, we started metabolic engineering of the mevalonate pathway at three different points: overproduction of Aat1, Idi1, and a truncated version of Hmg1, containing only the catalytic domain (Kampranis and Makris, 2012). In each case, the accumulation of lycopene was higher, supporting the accuracy of our pathway prediction. The most successful approach was transcriptional upregulation of *idi1*, indicating that this key step is also a clear bottleneck of the pathway in *U. maydis*. Idi1 is also known as the bottleneck in other systems like *S. cerevisiae*, where it was also initially tackled by *IDI1* overexpression or production of heterologous plant enzymes with better performances (Ignea et al., 2011; Ye et al., 2016). Multiple insertions of the *idi1* expression construct in *U. maydis*, however, did not increase lycopene levels further, suggesting that the *idi1* mRNA amount is no longer the limiting factor.

Production of the truncated version of Hmg1 was inspired by work in *S. cerevisiae*, where removing the N-terminal domain resulted in cytoplasmic localization due to detachment of the ER membrane and increased protein stability (Donald et al., 1997; Kampranis and Makris, 2012). Consistently, overproduction of Hmg1^N^*^Δ^*^1–932^ resulted in higher lycopene amounts. In this case, we did not obtain multiple insertions of the *hmg1*^N^*^Δ^*^1–932^ allele at the *ip*^s^ locus, suggesting that high levels of Hmg1^N^*^Δ^*^1–932^ cause growth defects. In the future, these alterations should be combined in a single production strain to further improve the performance of the underlying metabolic network.

Importantly, we verified the subcellular localization of the enzymes. Idi1 and the truncated Hmg1 version both localized in the cytoplasm as expected. But to our surprise Aat1 exhibited a peroxisomal localization, although no conventional peroxisomal targeting sequences were detectable. Peroxisomal targeting of acetoacetyl-CoA thiolases is reminiscent of the subcellular localization in mammalian and plant cells (Kovacs et al., 2007; Simkin et al., 2011). It was proposed in mammalian cells that the complete mevalonate pathway is occurring in peroxisomes (Kovacs et al., 2007). Since we observed that Idi1 appears to be cytoplasmic in *U. maydis*, the late steps of the pathway most likely take place in the cytoplasm. Thus, in basidiomycetes, the subcellular compartmentalization of the mevalonate pathway might be different. Interestingly, there are four additional enzymes annotated as putative acetoacetyl-CoA thiolases in the proteome of *U. maydis* (UMAG_03298, 01843, 01090, and 02715). In principle, these enzymes could be participating in the mevalonate pathway in the cytoplasm. In essence, successfully carrying out the initial steps in pathway engineering, we were able to lay a solid foundation for future improvements (see the section “Conclusion”).

### Production of Plant and Fungal Sesquiterpenoids in *U. maydis*

In order to demonstrate sesquiterpenoid production, we chose plant (+)-valencene and fungal α-cuprenene to be synthesized in *U. maydis*. (+)-valencene is extensively used in the flavor and fragrance industries, but also shows antagonistic activity against the plant pathogenic nematode *Heterodera schachtii* (Troost et al., 2019; Schleker et al., manuscript in preparation). Furthermore, it is a crucial intermediate for the synthesis of the higher value sesquiterpenoid nootkatone, which has a grapefruit-like odor and other bioactivities (Leonhardt and Berger, 2015). High (+)-valencene titers of 352 mg/L and 540 mg/L were reached in optimized *Rhodobacter sphaeroides* and *S. cerevisiae* strains, respectively (Beekwilder et al., 2014; Chen et al., 2019). Currently, a sustainable production process is marketed by the companies Evolva and Isobionics (now BASF) using the aforementioned microorganisms (Schempp et al., 2018; Chen et al., 2019). We therefore chose this target compound as a very prominent representative of heterologously produced sesquiterpenoids to benchmark our approach. Production of the plant (+)-valencene synthase CnVS in *U. maydis* alone resulted in (+)-valencene levels of 5.5 mg/L in shake flask fermentation, without extensive pathway engineering. To put it into perspective, heterologous expression of *CnVS* in *S. cerevisiae* resulted in initial titers of 1.3 mg/L (Cankar et al., 2014). Thus, our initial yield of (+)-valencene in *U. maydis* is a promising starting point but definitely needs further improvement (see the section “Conclusion”).

α-Cuprenene is originally synthesized by the sesquiterpenoid synthase Cop6 from *Coprinopsis cinerea*. The corresponding gene is part of a mini gene cluster flanked by *cox1* and *cox2* encoding cytochrome P450 monooxygenases. α-cuprenene is converted by these enzymes to lagopodin B that has antibacterial activity against Gram-positive bacteria (Agger et al., 2009; Stöckli et al., 2019). Interestingly, all members of the cluster are transcriptionally activated after co-cultivation with Gram-positive bacteria (Stöckli et al., 2019). We chose this target compound as a representative of basidiomycete speciality chemicals to test applicability of our approach. We succeeded in the production of α-cuprenene in *U. maydis* in reasonable amounts, reaching the estimated titer of 0.1 g/L in shake flask fermentation. Consistently, α-cuprenene was also produced in the basidiomycete yeast *X. dendrorhous*, using a similar strategy where Cop6 was produced to redirect FPP toward α-cuprenene. A comparable titer of 0.08 g/L was obtained after 4 days of cultivation and strains were able to produce α-cuprenene and astaxanthin simultaneously, indicating that, like in our case, the wild type FPP pool was not limited in sesquiterpenoid synthesis (Melillo et al., 2013).

In the future, Cop6 may be co-produced with the cytochrome P450 monooxygenases Cox1 and Cox2 for the synthesis of lagopodin B in *U. maydis* (Stöckli et al., 2019). Notably, the titer of the fungal compound was ten times higher than the plant compound. This is consistent with our initial hypothesis that the basidiomycete *U. maydis* might serve as a promising expression host for the production of sesquiterpenoids from higher basidiomycetes. In line with its potential application as novel production platform, *U. maydis* offers the clear advantage that genetic engineering is very well established (Brachmann et al., 2004; Terfrüchte et al., 2014). A comprehensive molecular tool box including, for example, six different resistance cassettes, regulatable promoters, as well as reporter genes like Gfp and glucuronidase are available (Brachmann et al., 2004; Terfrüchte et al., 2014). Homologous recombination is used for precise and stable integration of DNA constructs at defined genomic loci (Loubradou et al., 2001). Resistance marker recycling allows the generation of marker free production strains as well as the generation of multiple gene deletions (Khrunyk et al., 2010; Terfrüchte et al., 2018). Importantly, online bioengineering procedures for fermentation of *U. maydis* are already implemented (Kreyenschulte et al., 2015; Müller et al., 2018).

## Conclusion

We followed the microbial cell factory concept to establish the model basidiomycete fungus *U. maydis* as a novel chassis for the production of a broad range of terpenoids. As proof of concept, we have successfully produced two different sesquiterpenoids in *U. maydis* by modification of the existing mevalonate pathway and introduction of heterologous enzymes. In the future, additional genes encoding sesquiterpenoid synthases from higher basidiomycetes like *Omphalotus olearius* could be expressed to produce illudins and other compounds (Schmidt-Dannert, 2015; Xiao and Zhong, 2016).

To increase the yield of the desired compounds, there are several straightforward strategies conceivable that were successful in other microbial systems (Kampranis and Makris, 2012). Upregulation of the desired pathway would be possible by the production of heterologous enzymes with higher activity like optimized versions of bacterial Aat1 (Shiba et al., 2007). Competing pathways, such as that for ergosterol biosynthesis, could be reduced by downregulation of Erg9 squalene synthase production or by treatment with chemical inhibitors, which block the synthesis of ergosterol (Asadollahi et al., 2010). Alternatively, deletion of *fer4*, encoding an enoyl-CoA hydratase, will prevent the consumption of hydroxymethyl glutaryl-CoA during ferrichrome A synthesis (Winterberg et al., 2010). Finally, the fusion of FPP synthase (FPPS) and germacrene A synthase (GAS) has been shown to increase sesquiterpenoid yield, as FPP was directly funneled into product formation (Hu et al., 2017). Furthermore, in combination with the enhanced biomass degrading ability of *U. maydis* for use of alternative carbon sources (Geiser et al., 2016b; Stoffels et al., 2020), we envision to generate a sustainable consolidated bioprocessing strategy for next generation bioengineering of terpenoid production.

## Materials and Methods

### Bioinformatics Analysis

The KEGG database (Kyoto Encyclopedia of Genes and Genomes, Kanehisa and Goto, 2000; see text footnote 1) was used to identify candidates of the mevalonate pathway enzymes in *U. maydis* (Figure 1 and Table 1). For verification, amino acid sequences were compared to known enzymes from well-studied eukaryotes like *S. cerevisiae*, *H. sapiens*, and *A. thaliana* using BLASTP^2^ (Altschul et al., 1990). Clustal Omega and GeneDoc 2.6 were used for multiple amino acid sequence alignments and graphical representation (Supplementary Figure S1; Larkin et al., 2007). Domain structure was analyzed with SMART (Simple Modular Architecture Research Tool; analysis performed April 2020; Schultz et al., 1998; Letunic and Bork, 2018).

### Plasmids, Strains, and Growth Conditions

Standard molecular cloning procedures were carried out as published previously (Brachmann et al., 2004; Pohlmann et al., 2015). In brief, cloning was performed using *E. coli* K-12 derivate Top10 (Life Technologies, Carlsbad, CA, the USA) and standard techniques like Golden Gate as well as Gibson assembly were followed (Gibson et al., 2009; Terfrüchte et al., 2014). All *U. maydis* strains were generated by transformation of cells with linearized plasmids and homologous recombination events were verified by Southern blot analysis (see below; Brachmann et al., 2004).

The cultivation conditions and antibiotics used for *U. maydis* strains are described in Brachmann et al., 2004. In brief, complete medium was supplemented with 1% glucose (CM-glc) and strains were incubated at 28°C shaking in baffled flasks with 200 rpm. In order to induce the filamentous form of laboratory strain AB33, the medium was exchanged from CM-glc to nitrate minimal medium supplemented with 1% glucose (NM-glc). For monitoring cell growth and lycopene accumulation, a pre-culture was diluted to a starting OD_600_ of 0.25 in 500 mL volume non-baffled flask containing a total of 10 mL culture and incubated in white light (adapted from Estrada et al., 2009). Detailed information on plasmids and strains is given in Supplementary Tables S1–S3. Plasmid sequences are available on request.

Genomic DNA of wild type strain UM521 (*a1b1*) was used as template for PCR. Oligonucleotides used for molecular cloning are listed in Supplementary Table S4. For efficient expression of heterologous genes the codon usage was optimized using online tools from IDT (Integrated DNA Technologies, Leuven, Belgium) in the case of *Aa*CrtB^Myc^ or tailor-made context-dependent codon usage tools^3^ (Zarnack et al., 2006; Zhou et al., 2018) in the case of CnVS and Cop6. Cognate nucleotide sequences were chemically synthesized by IDT. Proteins were tagged with Gfp (enhanced green fluorescent protein; Clontech, Mountain View, CA, the USA) at their N-terminus unless noted otherwise. For recycling of resistance markers, the FLP-FRT system with different FRT site variants was applied as described (Khrunyk et al., 2010).

### Southern Blot Analysis

For Southern blot analysis genomic DNA (gDNA) of *U. maydis* strains was isolated and cleaved with appropriate restriction endonucleases (e.g., *Bam*HI in the case of insertion at the *ip*^s^ locus). The cleaved gDNA fragments were size-separated by electrophoresis (100 V, 3 h) on a 0.8% agarose gel. To break and depurinate large gDNA fragments, the gDNA-containing agarose gel was incubated in 0.25 M HCl and then further denatured and renatured in denaturation solution (1.5 M NaCl, 0.4 M NaOH) and renaturation solution (1.5 M NaCl, 0.28 M Tris-HCl, 0.22 M Tris-Base) for 20 min each. The gDNA fragments were transferred to a nylon membrane (Hybond-N^+^ nylon membrane, GE Healthcare, Chicago, IL, the USA) through a capillary blot using blotting solution (3.0 M NaCl, 0.3 M C_6_H_5_O_7_Na_3_^∗^2H_2_O, pH 7.0) for 12 h. After transfer the gDNA fragments were cross-linked to the membrane by UV irradiation (120 mJ/cm^2^, 254 nm, Biolink UV-Crosslinker, Vilber-Lourmat, Germany). The prehybridization step was carried out in hybridization buffer (780 mM NaCl, 59 mM NaHPO_4_^∗^H_2_O, 5.2 mM Na_2_-EDTA^∗^2H_2_O, pH 7.4; 0.1% [w/v] BSA fraction V, 0.1% [w/v] Ficoll 400, 0.1% [w/v] polyvinylpyrrolidone, 5% [w/v] SDS) at 65°C for 30 min. For detection of specific DNA fragments a DNA probe was labeled with digoxigenin (PCR Dig Labeling Mix, Roche, Mannheim, Germany). In the case of integration at the *ip*^s^ locus, a DNA fragment covering the open reading frame of the iron sulfur protein *ip*^s^ was amplified (888 base pair fragment; Loubradou et al., 2001). The double stranded probe was denatured in 20 mL of hybridization buffer at 98°C for 10 min. The membrane was hybridized with the denatured probe at 65°C for 12 h. To remove unbound probe, the membrane was incubated in 20 mL of membrane washing buffer I (300 mM NaCl, 22.7 mM NaHPO_4_^∗^H_2_O, 2 mM Na_2_-EDTA^∗^2H_2_O, pH 7.4, 0.1% [w/v] SDS), membrane washing buffer II (150 mM NaCl, 11.4 mM NaHPO_4_^∗^H_2_O, 1 mM Na_2_-EDTA^∗^2H_2_O, pH 7.4, 0.1% [w/v] SDS), and membrane washing buffer III (15 mM NaCl, 1.14 mM NaHPO_4_^∗^H_2_O, 0.1 mM Na_2_-EDTA^∗^2H_2_O, pH 7.4, 0.1% [w/v] SDS). Each washing step was carried out at 65°C for 15 min. Before adding α-DIG antibody (α-digoxigenin polyclonal antibody Fab fragments, Roche, Germany), which recognizes the digoxigenin-labeled probes, the membrane was incubated with DIG wash solution (0.3% [v/v] Tween-20 in DIG wash buffer I: 0.1 M maleic acid, 0.15 M NaCl, pH 7.5) at room temperature for 5 min. To reduce unspecific binding of the α-DIG antibody the membrane was blocked with DIG wash buffer II (1% [w/v] skimmed milk powder in DIG wash buffer I) at room temperature for 1 h. The α-DIG antibody was diluted (1:20,000) and incubated with the membrane at room temperature for 30 min. The membrane was washed twice in DIG wash solution for 15 min. Afterward the membrane was equilibrated at room temperature with DIG wash buffer III (0.1 M Tris-HCl, 0.1 M NaCl, pH 9.5) for 5 min. Subsequently, chemiluminescent substrate (1:100 CDP-Star, Roche, Germany, in DIG wash buffer III) was added to the membrane. Resulting chemiluminescence was detected with a LAS4000 ImageQuant device (GE Healthcare, the USA).

### Lycopene Extraction and Quantification

Lycopene extraction was adopted and modified as published (Ukibe et al., 2008). Cell pellets of a 10 mL culture after 48 h of incubation were washed in double-distilled water before disruption in acetone together with glass beads by shaking twice at a frequency of 30 Hz for 15 min in a pebble mill (MM400, Retsch GmbH, Haan, Germany). Afterwards, each suspension was heated for 10 min at 65°C to extract lycopene. The acetone was completely dried overnight at room temperature and samples were re-dissolved in 1 mL *n*-hexane. To remove cell debris, centrifugation at 16,000 × *g* for 10 min was performed twice. The lycopene extraction process was carried out at low light conditions.

A commercial reference of lycopene was purchased (SMB00706, Merck, Darmstadt, Germany) to compare absorbance wavelengths with lycopene extracts. Absorbance scanning was carried out in a UV-Vis spectrophotometer (Genesys 10S UV-Vis, Thermo Fisher Scientific, Waltham, MA, the USA) recording from 350 nm to 700 nm. For quantification of lycopene, absorption at 503 nm was determined and titer was assessed using molecular extinction coefficient 172,000 M^–1^cm^–1^ of lycopene in *n*-hexane (Fish et al., 2002).

### Protein Extraction and Western Blot Analysis

A culture of 20 mL growing to an OD_600_ of ca. 2 was harvested by centrifugation (16,000 × *g* for 10 min at room temperature). Cell pellets were frozen in liquid nitrogen and then disrupted in a pebble mill (MM400, Retsch GmbH) using a frequency of 30 Hz three times for one min with steal beads. Afterward, urea buffer (8 M urea, 50 mM Tris-HCl, pH 8) supplemented with protease inhibitors (one tablet of complete protease inhibitor per 20 mL; Roche, Germany, 0.1 M PMSF and 0.5 M benzamidine) was used for resuspension at 4°C. Cell debris was pelleted by centrifugation at 16,000 × *g* for 15 min at 4°C. The protein concentration in the supernatant was determined using a Bradford assay (Bio-Rad, Munich, Germany). 10% SDS-PAGE gels were used for separation of proteins, which were transferred to nitrocellulose membranes (Amersham Protran 0.45 NC Western blotting membrane, GE Healthcare Life Sciences, Munich, Germany) using a semi-dry Western blot procedure. Epitope-tagged proteins were detected using different primary antibodies produced in mouse, α-Gfp (monoclonal, Roche, Freiburg, Germany; 1:1,000 dilution), α-HA (monoclonal, Roche; 1:4,000 dilution), α-Myc (monoclonal, Merck; 1:5,000 dilution) and α-actin (monoclonal antibody raised against actin from chicken gizzard, MP Biomedicals, Eschwege, Germany; 1:500 dilution). Secondary α-mouse IgG-HRP conjugate (Promega, Mannheim, Germany; 1:4,000 dilution) was used for detection. The developing step was performed with Amersham ECL prime detection reagent and a LAS4000 chemi-luminescence imager (both GE Healthcare Life Sciences).

### Fluorimetric Measurements

A pre-culture was diluted to an OD_600_ 0.5 in CM-glc for all strains and 1 mL of each culture was harvested at 16,000 × *g* for 5 min at room temperature. The cell pellets were washed twice in double-distilled water. Afterward, each cell pellet was resuspended in 1 mL of double-distilled water. 200 μL of each sample was transferred into black 96-well plates (Greiner Bio-One, Frickenhausen, Germany) for measurements in an Infinite M200 plate reader (Tecan Group Ltd., Männedorf, Switzerland). As a blank 200 μL of double-distilled water was used. Within the microplate reader, measurements of OD_600_ and fluorescence intensity were performed. In the case of Gfp, an excitation wavelength of 483 nm and an emission wavelength of 535 nm were used. At least three independent biological experiments were performed with three technical replicates per strain.

### Fluorescence Microscopy

To visualize the nucleus, cells were first fixed with 1% formaldehyde for 30 min and washed twice in PBS. Afterward, the DNA was stained with Hoechst 33342 dye (H1399, Thermo Fisher Scientific). In brief, 10 mg/mL stock solution was diluted to 1:2000 in PBS and the fixed cells were stained for 10 min at room temperature. Excess dye was washed three times with PBS.

Microscopy was carried out as described before (Baumann et al., 2016; Jankowski et al., 2019) using two systems: (i) a wide-field microscope set-up from Visitron Systems (Puchheim, Germany), Axio Imager M1 equipped with a Spot Pursuit CCD camera (Diagnostic Instruments, Sterling Heights, MI, the USA) and the objective lens Plan Neofluar (40×, NA 1.3; 63×, NA 1.25; Carl Zeiss, Jena, Germany). The excitation of fluorescently labeled proteins was carried out using an HXP metal halide lamp (LEj, Jena, Germany) in combination with a filter set for green fluorescent protein (ET470/40BP, ET495LP and ET525/50BP) and Hoechst 33342 dye (HC387/11BP, BS409LP and HC 447/60BP; AHF Analysentechnik, Tübingen, Germany). The microscopic system was controlled by MetaMorph software (Molecular Devices, version 7, Sunnyvale, CA, the USA). The program was also used for image processing, including the adjustment of brightness and contrast.

(ii) In order to record fluorescence signals localized in subcellular compartments with higher sensitivity, laser-based epifluorescence microscopy was performed on a Zeiss Axio Observer.Z1 equipped with CoolSNAP HQ2 CCD (Photometrics, Tuscon, AZ, the USA) and ORCA-Flash4.0 V2 + CMOS (Hamamatsu Photonics Deutschland GmbH, Geldern, Germany) cameras. The microscopy set-up was the same as described above (Jankowski et al., 2019). For excitation, a VS-LMS4 Laser-Merge-System (Visitron Systems) that combines solid-state lasers for excitation of Gfp (488 nm at 50 or 100 mW) was used. Videos were recorded with an exposure time of 150 ms and 150 frames were taken. All videos and images were processed and analyzed using MetaMorph (Version 7.7.0.0, Molecular Devices). Kymographs were generated using a built-in plugin.

### Analysis of Sesquiterpenoids Using GC-MS

For the analysis of (+)-valencene and α-cuprenene, 10 mL of cells grown to an OD_600_ of approximately 6 were harvested after 48 h. To trap the secreted sesquiterpenoids, 500 μL of *n*-dodecane (D0968, TCI Deutschland GmbH, Eschborn, Germany) were added to the shaking culture. To ensure phase separation, the *n*-dodecane containing layer was centrifuged twice at 16,000 × *g* for 10 min. The *n*-dodecane phase-containing sesquiterpenoids were analyzed using a GC/MS-QP2010 (Shimadzu, Tokyo, Japan) equipped with a FS-Supreme-5 column (30 m × 0.25 mm × 0.25 μm; Chromatographie Service GmbH, Langerwehe, Germany). The GC-MS conditions were adopted from a previous study (Schulz et al., 2015). Temperatures of the injector and interface were set at 250°C and 285°C, respectively. The carrier gas was helium and its velocity was set to 30 cm sec^–1^. 1 μL of the sample was injected with a split ratio of 10. The column temperature was sequentially changed and maintained at 130°C for 3 min, ramped to 260°C at a rate of 10°C min^–1^, held at 260°C for 1 min, ramped to 300°C at a rate of 40°C min^–1^ and held at 300°C for 1 min. In the case of (+)-valencene, a purchased reference compound (75056, Merck) was diluted as 50 μM and 200 μM in *n*-dodecane samples of the negative control. The retention time and fragmentation pattern of the mass spectrum obtained with the *U. maydis* sample were compared with the reference. In addition, the fragmentation patterns of (+)-valencene and α-cuprenene were compared with the previously reported data (Agger et al., 2009; Troost et al., 2019).

### Quantification of Sesquiterpenoid Production With GC-FID

In order to determine product titers of (+)-valencene and α-cuprenene, *n*-dodecane samples were subjected to the Agilent 6890N gas chromatograph equipped with a (5%-phenyl)-methylpolysiloxane HP-5 column (length, 30 m; inside diameter, 0.32 mm; film thickness, 0.25 mm; Agilent Technologies, Ratingen, Germany) and a flame ionization detector (FID). Both heterologously produced sesquiterpenoid samples and standard calibration samples were diluted in ethyl acetate (Rodriguez et al., 2014). Temperatures of the injector and FID were set to 240°C and 250°C, respectively. Each sample had a volume of 1 μL and was injected splitless with helium as a carrier gas. The column temperature was sequentially changed: starting at 100°C for 5 min, ramped at 10°C min^–1^ to 180°C and then at 20°C min^–1^ to 300°C. The signals of heterologously produced (+)-valencene, which were absent in control samples, were confirmed by comparison of retention time to a commercial reference of (+)-valencene (75056, Merck). To estimate the titer of α-cuprenene, the chemically similar reference compound β-caryophyllene was used (22075, Merck), because α-cuprenene is not commercially available to correlate putative α-cuprenene signals, which were absent in control samples, to compound amounts.

## Data Availability Statement

All datasets presented in this study are included in the article/Supplementary Material.

## Author Contributions

JL, AL, K-EJ, and MF designed and planned the study. JL performed the experiments. FH and AL supported GC-FID analysis. JL, FH, AL, K-EJ, and MF analyzed the data. JL and MF designed and revised the manuscript. MF directed the project. All authors contributed to the article and approved the submitted version.

## Conflict of Interest

The authors declare that the research was conducted in the absence of any commercial or financial relationships that could be construed as a potential conflict of interest.

## Footnotes

1 https://www.genome.jp/kegg/

2 https://blast.ncbi.nlm.nih.gov/blast/

3 http://dicodon-optimization.appspot.com
