# Supplementary material for: Ustilago maydis Serves as a Novel Production Host for the Synthesis of Plant and Fungal Sesquiterpenoids
Source: Front Microbiol. 2020 Jul 24;11:1655. doi: 10.3389/fmicb.2020.01655 (PMC7396576; doi:10.3389/fmicb.2020.01655)
Supplement: Supplementary file 1 [file Data_Sheet_1.PDF]

Supplementary Material of the manuscript entitled

Ustilago maydis serves as a novel production host for the synthesis of plant and fungal sesquiterpenoids

Jungho Lee[1], Fabienne Hilgers[2,3], Anita Loeschke[2,3], Karl-Erich Jaeger[2,3] and Michael Feldbrügge[1,*]

Including Supplementary Figure S1-4 as well as Supplementary Table S1-S4

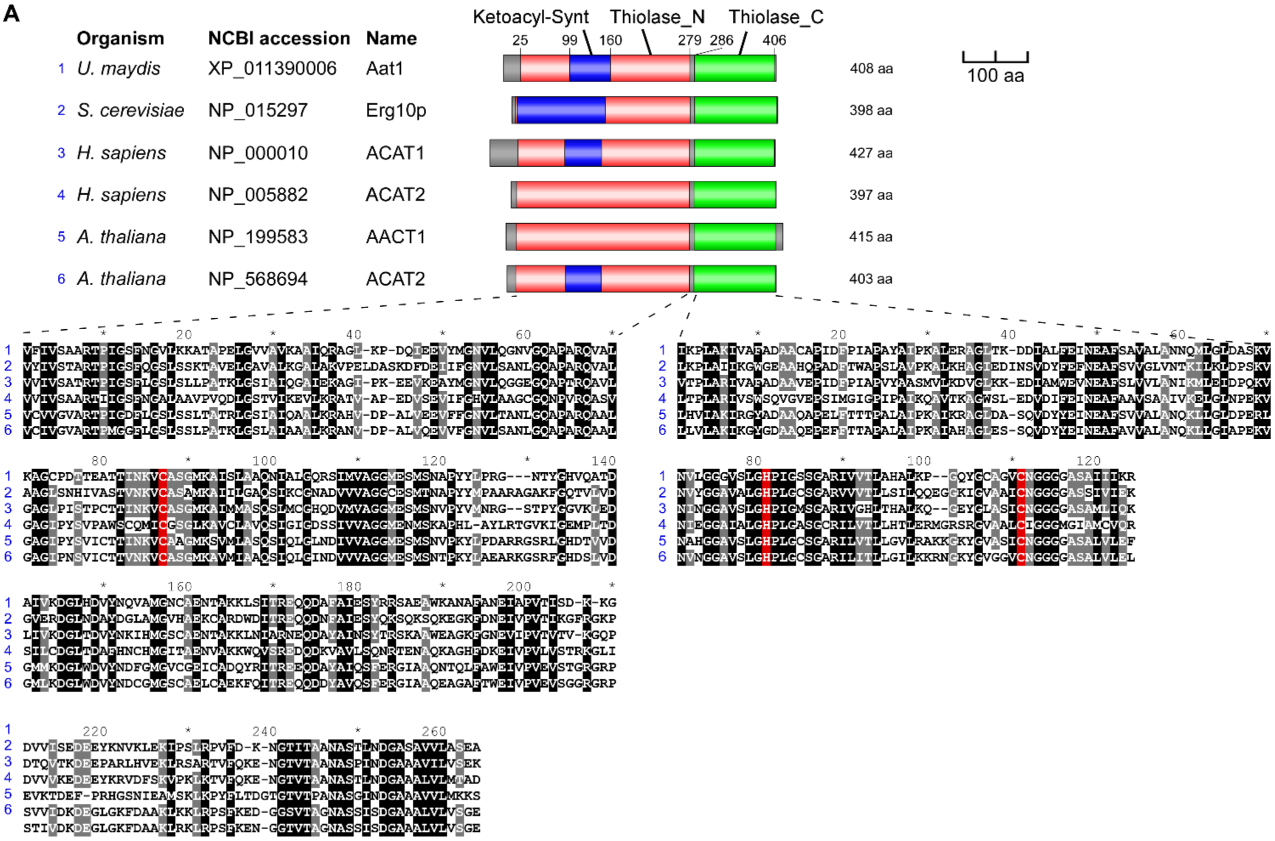

Supplementary Figure S1. Bioinformatics analysis of *U. maydis* biosynthetic enzymes. (**A**) Amino acid sequence comparison of acetyl-CoA C-acetyltransferase Aat1 with homologues in fungi, animals and plants. Domain architecture according to SMART is given on the top (Ketoacyl-Synt, protein families Pfam identifier PF00109; Thiolase_N, PF00108; Thiolase_C, PF02803) and a sequence alignment of the corresponding domains is given at the bottom. Key amino acids in the active site of the enzyme from the bacterium *Zoogloea ramigera* are given in red (Miziorko, 2011). (**B-H**) Continued on next page.

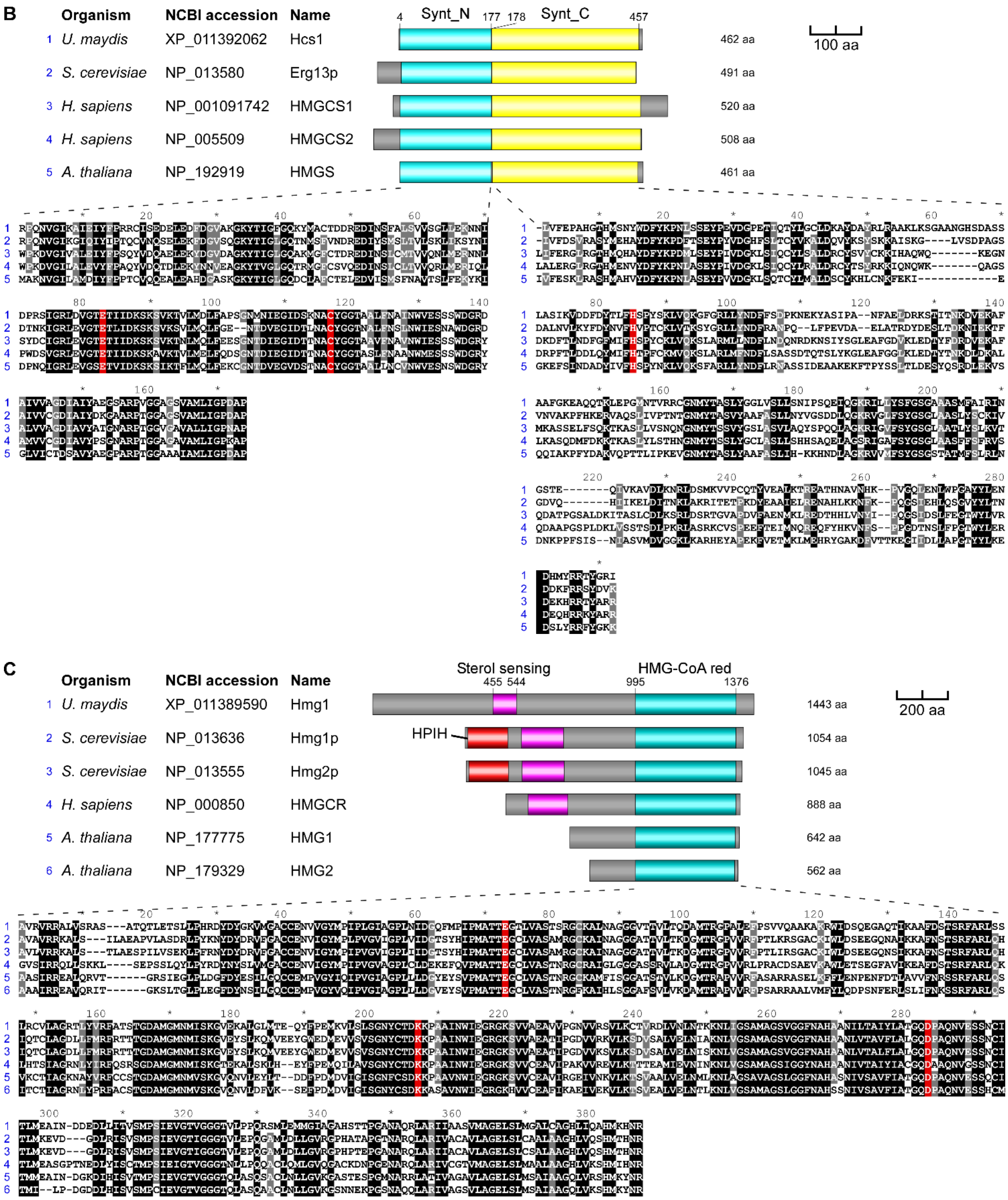

Supplementary Figure S2. Bioinformatics analysis of *U. maydis* biosynthetic enzymes. (**B-C**) Amino acid sequence comparison of 3-hydroxy-3-methylglutaryl-CoA synthases (B, active site from *Staphylococcus aureus*) and 3-hydroxy-3-methylglutaryl-CoA reductases (C, active site from *H. sapiens*) as shown in (A) (Miziorko, 2011; Synt_N, PF01154; Synt_C, PF08540; Sterol sensing, PF12349; HMG-CoA red, PF00368; HPIH, PF13323). (**D-H**) Continued on next page.

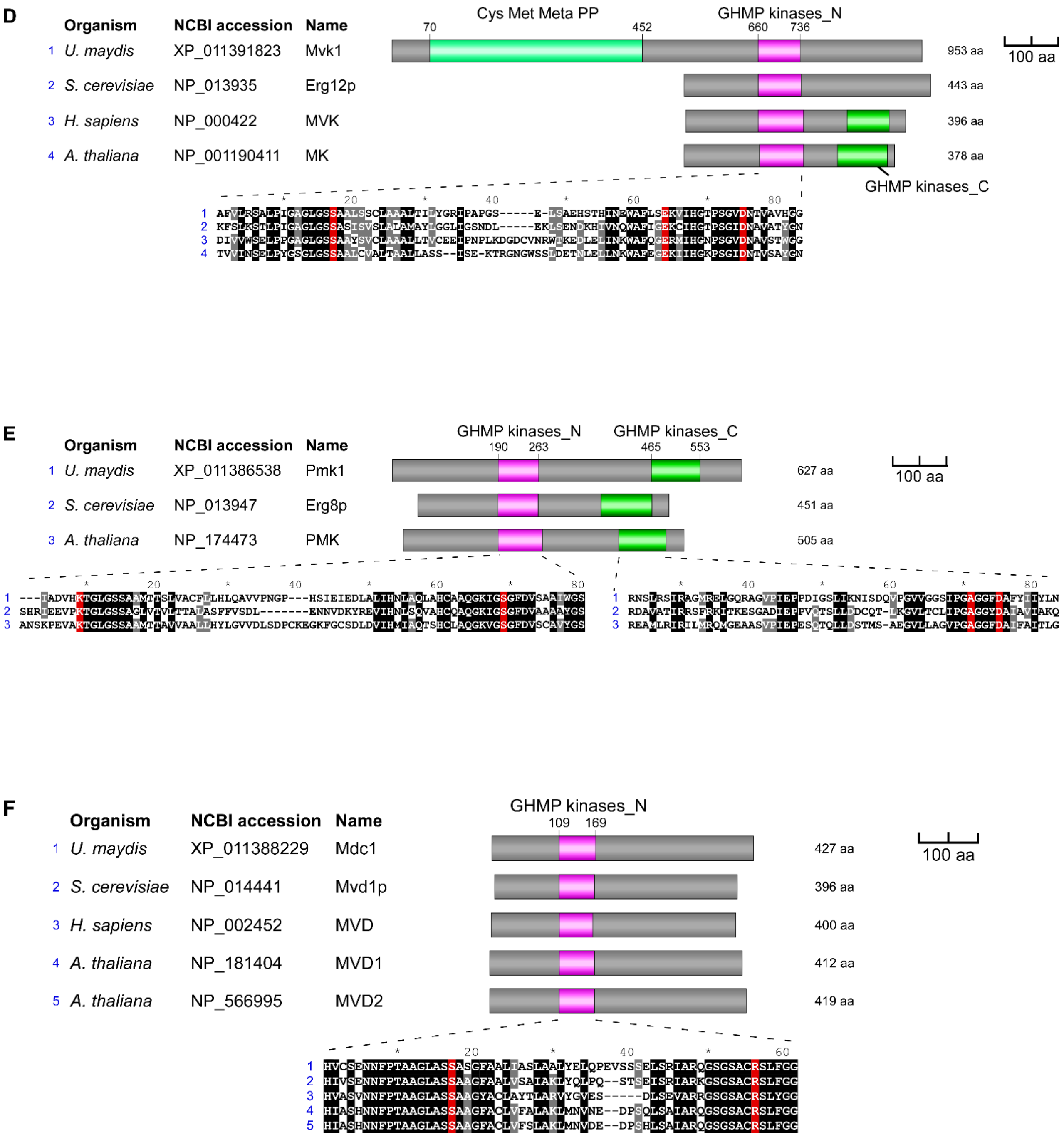

Supplementary Figure S3. Bioinformatics analysis of *U. maydis* biosynthetic enzymes.

(**D-F**) Amino acid sequence comparison of mevalonate kinases (D, active site from *Rattus norvegicus*) and phosphomevalonate kinases (E, active site from the bacterium *Streptococcus pneumoniae*, Andreassi et al. 2009 Biochemistry 48:6461) and mevalonate diphosphate decarboxylases (F, active site from *H. sapiens*) as shown in (A) (Miziorko, 2011; Cys Met Meta PP, PF01053; GHMP kinases_N, PF00288; GHMP kinases_C, PF08544). (**G-H**) Continued on next page.

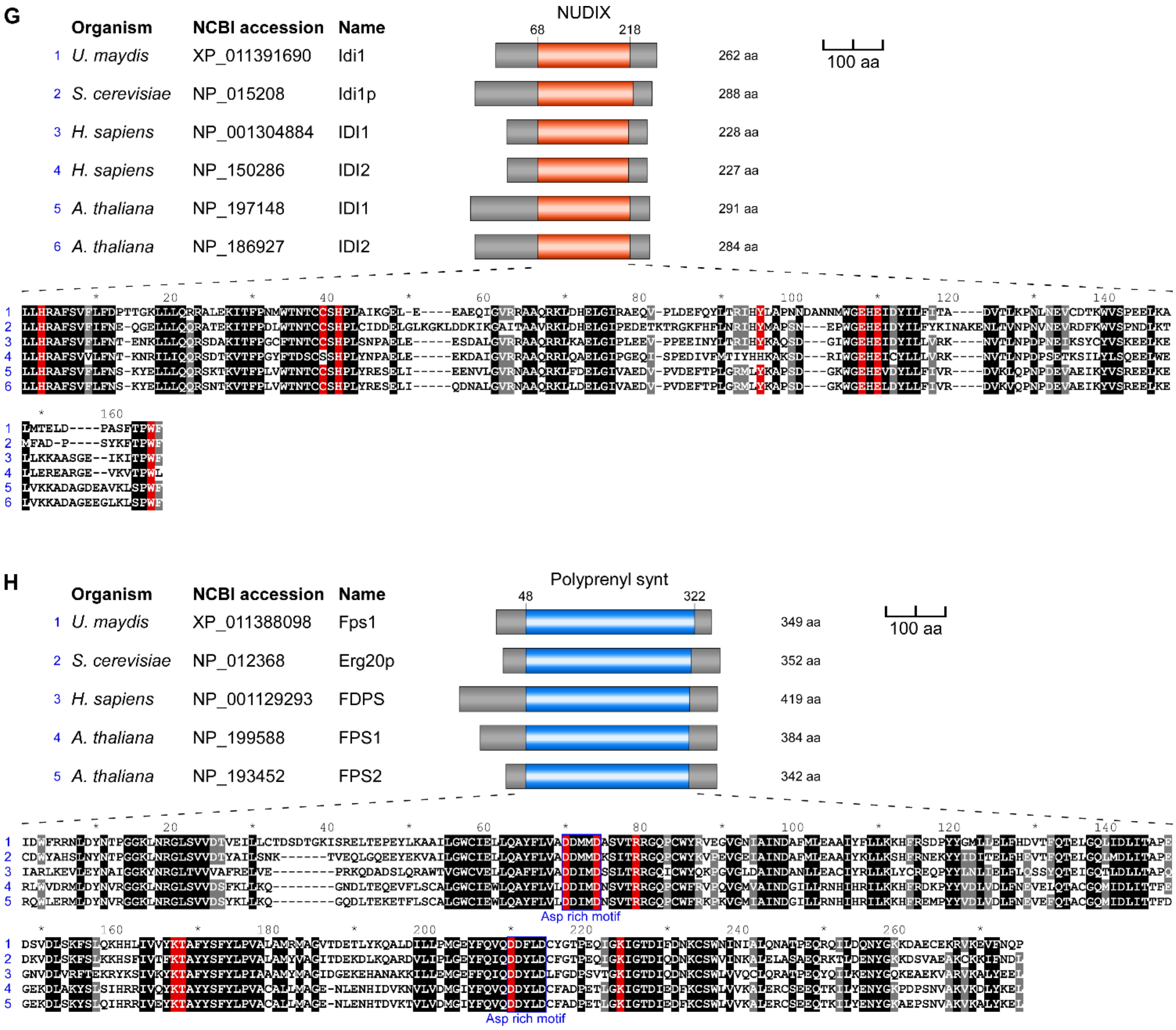

Supplementary Figure S4. Bioinformatics analysis of *U. maydis* biosynthetic enzymes.

(**G-H**) Amino acid sequence comparison of isopentenyl diphosphate isomerases (G, active site from *H. sapiens;* Zheng et al. 2007 *J. Mol. Biol.* 366:1447) and farnesyl diphosphate synthase/dimethylallyl transtransferases (H, active site from *H. sapiens*; Kavanagh et al. 2006 *PNAS* 103:7829) as shown in (A; NUDIX, PF00293; Polyprenyl synt, PF00348). End of figure.

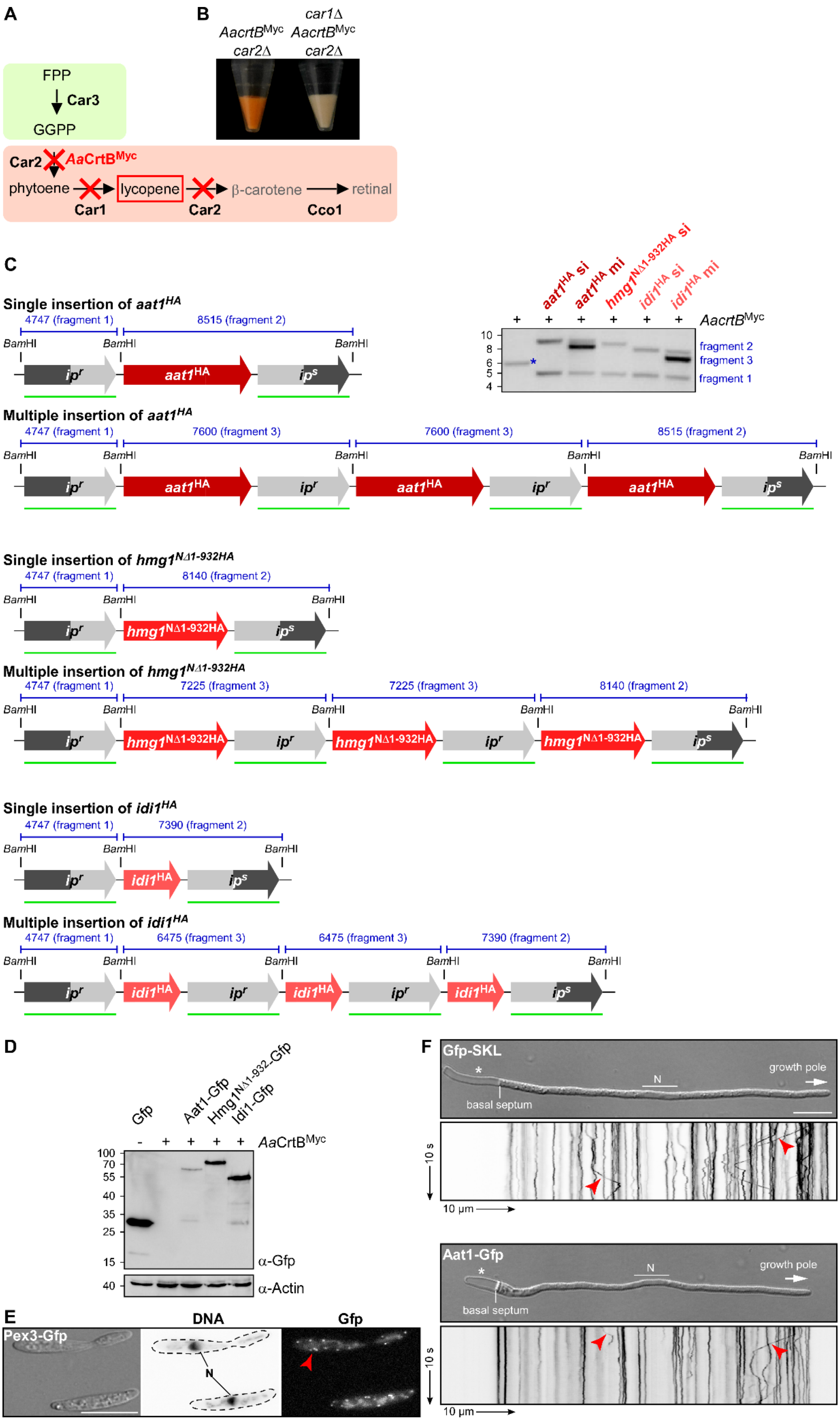

Supplementary Figure S2. Aat1 localises to motile peroxisomes during hyphal growth.

(**A**) Schematic representation of the carotenoid module given in Figure 1 (red cross indicates gene deletion). (**B**) Cell pellets of strains indicated above the image. (**C**) Graphical representation of the genetic modification at the ip^s locus encoding an iron sulfur protein conferring carboxin resistance. The expected gene structure for *aat1*^{HA}, *hmg1*$^{N\Delta 1\text{-}932HA}$ and *idi1*^{HA} are given. Single and multiple insertions are compared. For the sake of clarity, the Southern Blot shown in Figure 3B is given in the right upper corner. The wild type (*wt*) version encoding a sensitive version ip^s is given in dark grey. The corresponding resistant version is given in light grey (ip^r). The expected fragments are given in blue. Note, that due to the repetition during multiple insertion, the intensity of fragment 3 is always the strongest. The probe used for hybridisation is indicated as a green line. (**D**) Western blot analysis of strains indicated above the lanes. The expected molecular weight is 70 kDa for Aat1-Gfp, 81 kDa for Hmg1$^{N\Delta 1\text{-}932}$-Gfp, 57 kDa for Idi1-Gfp, 37 kDa for *Aa*CrtB^{Myc} and 57 kDa for actin (UMAG_11232). Antibodies are given in the lower right corner (size marker in kDa given on the left). Note, that the *Aa*CrtB^{Myc} producing strains carried a deletion of *car2*. (**E**) Microscopic analysis showing DIC image of fixed cells on the left (size bar, 10 µm). Corresponding staining of nuclear DNA with Hoechst 33342 (middle panel; N, nucleus, inverted image) and green fluorescence (Gfp) on the right (red arrowheads indicate peroxisomes). (**F**) Microscopic analysis showing DIC images of AB33 hyphae (6 h.p.i.) on top (size bar, 10 µm; N, nucleus) and corresponding kymographs at the bottom; genetic background as indicated (arrow length on the left and bottom indicates time and distance, respectively). Bidirectional movement of peroxisomes is visible as diagonal lines (red arrowheads).

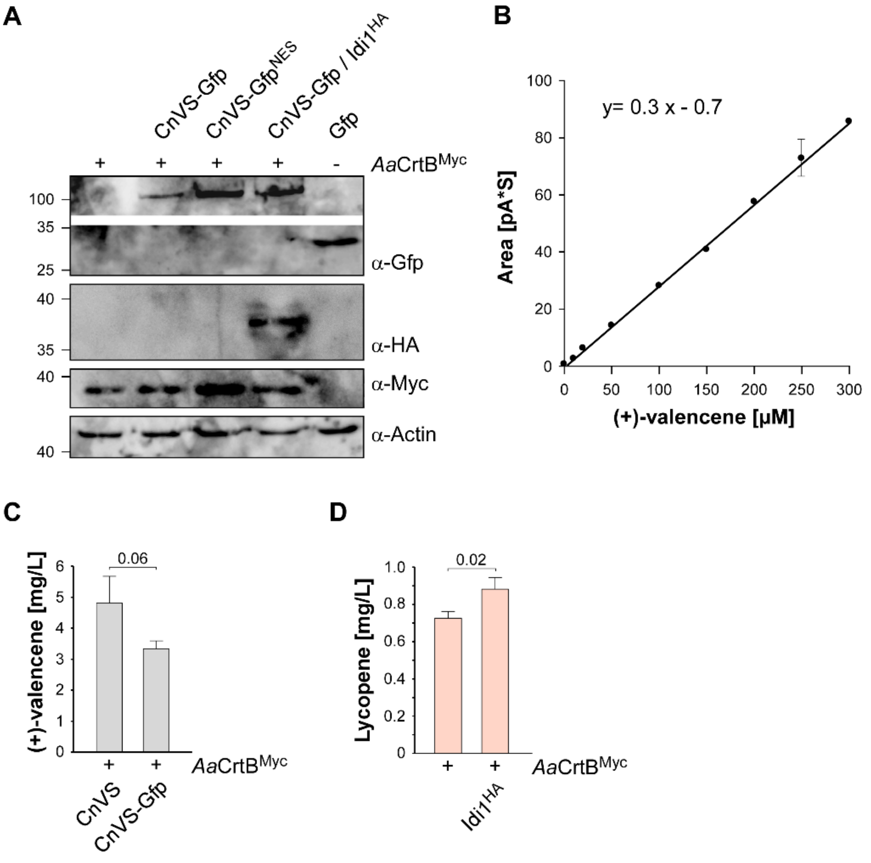

Supplementary Figure S3. Production of (+)-valencene synthase CnVS in *U. maydis*.

(**A**) Western blot analysis of strains indicated above the lanes. The expected molecular weight is 96 kDa for CnVS-Gfp, 97 kDa for CnVS-Gfp^{NES}, 27 kDa for Gfp, 37 kDa for *Aa*CrtB^{Myc} and 42 kDa for actin (UMAG_11232). Antibodies are given in the lower right corner (size marker in kDa given on the left). Note, that the *Aa*CrtB^{Myc} producing strain carried a deletion of *car2*. (**B**) Standard calibration curve of the peaks derived from GC-FID measurements versus (+)-valencene concentrations. (**C**) Concentrations of (+)-valencene in the *n*-dodecane samples were determined based on the calibration curve with the commercial reference compound used in (B). Three independent biological experiments (n=3) were carried out. Error bars indicate standard deviation of the mean (SD). Statistical significance was calculated using the unpaired two-tailed *t* test and *p*-values were indicated above. Note, that the *Aa*CrtB^{Myc} producing strains carried a deletion of *car2*. (**D**) Lycopene concentrations in strains given at the bottom. Three independent biological experiments (n=3) were carried out. Error bars indicate standard deviation of the mean (SD). Statistical significance was calculated using the unpaired two-tailed *t* test and *p*-values were indicated above. Note, that the *Aa*CrtB^{Myc} producing strains carried a deletion of *car2*.

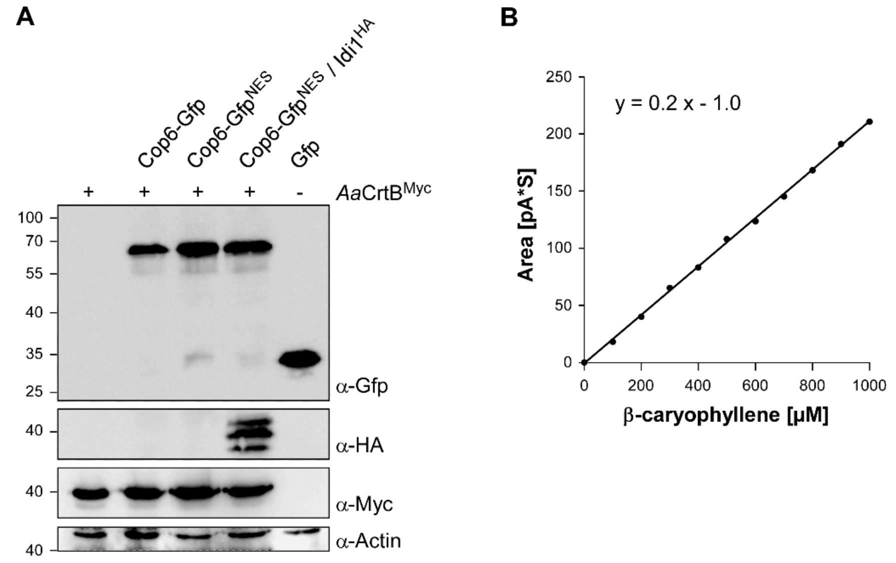

Supplementary Figure S4. Heterologous production of Cop6 in *U. maydis*.

(**A**) Western blot analysis of strains indicated above the lanes. The expected molecular weight is 65 kDa for Cop6-Gfp, 66 kDa for Cop6-Gfp^{NES}, 27 kDa for Gfp, 37 kDa for *Aa*CrtB^{Myc} and 42 kDa for actin (UMAG_11232). Antibodies are given in the lower right corner (size marker in kDa given on the left). Note, that the *Aa*CrtB^{Myc} producing strains carried a deletion of *car2*. (**B**) Standard calibration curve of the peaks derived from GC-FID measurements versus the chemically similar compound β-caryophyllene.

Supplementary Table S1: Description of *U. maydis* strains used in this study

Strain	Locus	Progenitor strain	Short description
AB33	*b*	FB2	*Pnar:bW2bE1*, production of active *b* heterodimer under control of the P$_{nar1}$ promoter; strain grows filamentously upon changing the nitrogen source.<br>Published in (Brachmann, 2001).
AB33car2Δ_HygR	*car2*	AB33	Carrying a deletion of *car2* and possessing hygromycin B resistance.
AB33car2Δ/AacrtB^{Myc}_NatR	*car2*	AB33car2Δ_HygR	Carrying a deletion of *car2* and producing codon-optimized *Aa*CrtB, which is C-terminally fused to 3× Myc tag at the *car2* locus. Possessing nourseothricin resistance.
AB33car2Δ/AacrtB^{Myc}_NatR/<br>car1Δ_HygR	*car2*<br>*car1*	AB33car2Δ/AacrtB^{Myc}_NatR	Carrying deletions of *car2* and *car1* and producing codon-optimized *Aa*CrtB, which is C-terminally fused to 3× Myc tag at the *car2* locus. Possessing nourseothricin and hygromycin B resistance.
AB33car2Δ/AacrtB^{Myc}_NatR/<br>aat1^{HA}_CbxR	*car2*<br>*ip*^{s}	AB33car2Δ/AacrtB^{Myc}_NatR	Carrying a deletion of *car2* and co-producing codon-optimized *Aa*CrtB, which is C-terminally fused to 3× Myc tag at the *car2* locus, and Aat1 N-terminally fused to 3× HA tag at the *ip*^{s} locus. Possessing nourseothricin and carboxin resistance.
AB33car2Δ/AacrtB^{Myc}_NatR/<br>hmg1$^{N\Delta 1\text{-}932HA}$_CbxR	*car2*<br>*ip*^{s}	AB33car2Δ/AacrtB^{Myc}_NatR	Carrying a deletion of *car2* and co-producing codon-optimized *Aa*CrtB, which is C-terminally fused to 3× Myc tag at the *car2* locus, and N-terminally truncated from aa 1 to 932 version of Hmg1, which is N-terminally fused to 3× HA tag at the *ip*^{s} locus. Possessing nourseothricin and carboxin resistance.
AB33car2Δ/AacrtB^{Myc}_NatR/<br>idi1^{HA}_CbxR	*car2*<br>*ip*^{s}	AB33car2Δ/AacrtB^{Myc}_NatR	Carrying a deletion of *car2* and co-producing codon-optimized *Aa*CrtB, which is C-terminally fused to 3× Myc tag at the *car2* locus, and Idi1 N-terminally fused to 3× HA tag at the *ip*^{s} locus. possessing nourseothricin and carboxin resistance.
AB33car2Δ/AacrtB^{Myc}_NatR/<br>aat1G_CbxR	*car2*<br>*ip*^{s}	AB33car2Δ/AacrtB^{Myc}_NatR	Carrying a deletion of *car2* and co-producing codon-optimized *Aa*CrtB, which is C-terminally fused to 3× Myc tag at the *car2* locus, and Aat1 N-terminally fused to eGfp at the *ip*^{s} locus. Possessing nourseothricin and carboxin resistance.
AB33car2Δ/AacrtB^{Myc}_NatR/<br>hmg1$^{N\Delta 1\text{-}932}$G_CbxR	*car2*<br>*ip*^{s}	AB33car2Δ/AacrtB^{Myc}_NatR	Carrying a deletion of *car2* and co-producing codon-optimized *Aa*CrtB, which is C-terminally fused to 3× Myc tag at the *car2* locus, and N-terminally truncated from aa 1 to 932 version of Hmg1, which is N-terminally fused to eGfp at the *ip*^{s} locus. Possessing nourseothricin and carboxin resistance.
AB33car2Δ/AacrtB^{Myc}_NatR/<br>idi1G_CbxR	*car2*<br>*ip*^{s}	AB33car2Δ/AacrtB^{Myc}_NatR	Carrying a deletion of *car2* and co-producing codon-optimized *Aa*CrtB, which is C-terminally fused to 3× Myc tag at the *car2* locus and Idi1 N-terminally fused to eGfp at the *ip*^{s} locus. Possessing nourseothricin and carboxin resistance.
AB33egfp_CbxR	*ip*^{s}	AB33	The *egfp* construct is ectopically integrated into the *ip*^{s} locus. Possessing nourseothricin and carboxin resistance. Published in (Köpke, 2011).
AB33upa9mC_HygR/<br>egfp-skl_CbxR	*upa9*<br>*ip*^{s}	AB33egfp-skl_CbxR	Co-producing eGfp containing SKL at the C-terminus at the *ip*^{s} locus and Upa9 C-terminally fused to eGfp at the *upa9* locus. Upa9 is the homolog of Pex3.
AB33pab1mC_HygR/<br>upa9G_NatR	*pab1*<br>*upa9*	AB33pab1mC	Co-producing Pab1 C-terminally fused to mCherry at the *pab1* locus and Upa9 C-terminally fused to eGfp at the *upa9* locus. Possessing hygromycin B and carboxin resistance.
AB33car2Δ/AacrtB^{Myc}	*car2*	AB33car2Δ/AacrtB^{Myc}_NatR	Carrying a deletion of *car2* and producing codon-optimized *Aa*CrtB, which is C-terminally fused to 3× Myc tag at the *car2* locus. Nourseothricin resistance gene cassette is recycled via FRTm7
AB33car2Δ/AacrtB^{Myc}/<br>cco1Δ_HygR	*car2*<br>*cco1*	AB33car2Δ/AacrtB^{Myc}	Carrying deletions of *car2* and *cco1* and producing codon-optimized *Aa*CrtB, which is C-terminally fused to 3× Myc tag at the *car2* locus. Nourseothricin resistance gene cassette is recycled via FRTm7 and possessing hygromycin B resistance.
AB33car2Δ/AacrtB^{Myc}/cco1Δ/<br>idi1^{HA}_NatR	*car2*<br>*cco1*	AB33car2Δ/AacrtB^{Myc}/cco1Δ<br>_HygR	Carrying deletions of *car2* and *cco1* and co-producing codon-optimized *Aa*CrtB, which is C-terminally fused to 3× Myc tag at the *car2* locus, and Idi1 N-terminally fused to 3× HA tag at the *cco1* locus. Possessing nourseothricin resistance.

AB33car2Δ/AacrtB^{Myc}/cco1Δ/ idi1^{HA}	*car2* *cco1*	AB33car2Δ/AacrtB^{Myc}/cco1Δ /idi1^{HA}_NatR	Carrying deletions of *car2* and *cco1* and co-producing codon-optimized *Aa*CrtB, which is C-terminally fused to 3× Myc tag at the *car2* locus, and Idi1 N-terminally fused to 3× HA tag at the *cco1* locus. Nourseothricin resistance gene cassette is recycled via FRTm1.
AB33car2Δ/AacrtB^{Myc}/cco1Δ/ idi1^{HA}/upp3Δ_HygR	*car2* *cco1*	AB33car2Δ/AacrtB^{Myc}/cco1Δ /idi1^{HA}	Carrying deletions of *car2*, *cco1*, and *upp3*, and co-producing codon-optimized *Aa*CrtB, which is C-terminally fused to 3× Myc tag at the *car2* locus, and Idi1 N-terminally fused to 3× HA tag at the *cco1* locus. Possessing hygromycin B resistance.
AB33upp3Δ/egfp_NatR	*upp3*	AB33upp3Δ_HygR	Carrying a deletion of *upp3* and producing eGfp at the *upp3* locus. Possessing nourseothricin resistance.
AB33car2Δ/AacrtB^{Myc}/ upp3Δ_HygR	*car2* *upp3*	AB33car2Δ/AacrtB^{Myc}	Carrying deletions of *car2* and *upp3* and producing codon-optimized *Aa*CrtB, which is C-terminally fused to 3× Myc tag at the *car2* locus. Possessing hygromycin B resistance
AB33car2Δ/AacrtB^{Myc}/upp3Δ/ CnVS_NatR	*car2* *upp3*	AB33car2Δ/AacrtB^{Myc}/upp3Δ _HygR	Carrying deletions of *car2* and *upp3* and co-producing codon-optimized *Aa*CrtB, which is C-terminally fused to 3× Myc tag at the *car2* locus, and dicodon-optimized CnVS at the *upp3* locus. Possessing nourseothricin resistance.
AB33car2Δ/AacrtB^{Myc}/upp3Δ/ CnVSG_NatR	*car2* *upp3*	AB33car2Δ/AacrtB^{Myc}/upp3Δ _HygR	Carrying deletions of *car2* and *upp3* and co-producing codon-optimized *Aa*CrtB, which is C-terminally fused to 3× Myc tag at the *car2* locus, and dicodon-optimized CnVS, which is N-terminally fused to eGfp at the *upp3* locus. Possessing nourseothricin resistance.
AB33car2Δ/AacrtB^{Myc}/upp3Δ/ CnVSG^{NES}_NatR	*car2* *upp3*	AB33car2Δ/AacrtB^{Myc}/upp3Δ _HygR	Carrying deletions of *car2* and *upp3* and co-producing codon-optimized *Aa*CrtB, which is C-terminally fused to 3× Myc tag at the *car2* locus, and dicodon-optimized CnVS, which is N-terminally fused to eGfp^{NES} at the *upp3* locus. Possessing nourseothricin resistance.
AB33car2Δ/AacrtB^{Myc}/cco1Δ/ idi1^{HA}/upp3Δ/CnVSG^{NES}_NatR	*car2* *cco1* *upp3*	AB33car2Δ/AacrtB^{Myc}/cco1Δ /idi1^{HA}/upp3Δ_HygR	Carrying deletions of *car2*, *cco1*, and *upp3* and co-producing codon-optimized *Aa*CrtB, which is C-terminally fused to 3× Myc tag at the *car2* locus, Idi1 N-terminally fused to 3× HA tag at the *cco1* locus, and dicodon-optimized CnVS, which is N-terminally fused to eGfp^{NES} at the *upp3* locus. Possessing nourseothricin resistance.
AB33car2Δ/AacrtB^{Myc}/upp3Δ/ cop6_NatR	*car2* *upp3*	AB33car2Δ/AacrtB^{Myc}/upp3Δ _HygR	Carrying deletions of *car2* and *upp3* and co-producing codon-optimized *Aa*CrtB, which is C-terminally fused to 3× Myc tag at the *car2* locus, and dicodon-optimized Cop6 at the *upp3* locus. Possessing nourseothricin resistance.
AB33car2Δ/AacrtB^{Myc}/upp3Δ/ cop6G_NatR	*car2* *upp3*	AB33car2Δ/AacrtB^{Myc}/upp3Δ _HygR	Carrying deletions of *car2* and *upp3* and co-producing codon-optimized *Aa*CrtB, which is C-terminally fused to 3× Myc tag at the *car2* locus, and dicodon-optimized Cop6, which is N-terminally fused to eGfp at the *upp3* locus. Possessing nourseothricin resistance.
AB33car2Δ/AacrtB^{Myc}/upp3Δ/ cop6G^{NES}_NatR	*car2* *upp3*	AB33car2Δ/AacrtB^{Myc}/upp3Δ _HygR	Carrying deletions of *car2* and *upp3* and co-producing codon-optimized *Aa*CrtB, which is C-terminally fused to 3× Myc tag at the *car2* locus and dicodon-optimized Cop6, which is N-terminally fused to eGfp^{NES} at the *upp3* locus. Possessing nourseothricin resistance.
AB33car2Δ/AacrtB^{Myc}/cco1Δ/ idi1^{HA}/upp3Δ/cop6G^{NES}_NatR	*car2* *cco1* *upp3*	AB33car2Δ/AacrtB^{Myc}/cco1Δ /idi1^{HA}/upp3Δ_HygR	Carrying deletions of *car2*, *cco1*, and *upp3* and co-producing codon-optimized *Aa*CrtB, which is C-terminally fused to 3× Myc tag at the *car2* locus, Idi1 N-terminally fused to 3× HA tag at the *cco1* locus, and dicodon-optimized Cop6, which is N-terminally fused to eGfp^{NES} at the *upp3* locus. Possessing nourseothricin resistance.

Supplementary Table S2: Generation of *U. maydis* strains used in this study

Strain	Relevant genotype	UMa	Reference	Transformed plasmid (pUMa)	Locus	Progenitor strain
AB33	*a2 Pnar::bW2bE1*	133	Brachmann, 2001	pAB33	*b*	FB2
AB33car2Δ_HygR	*car2Δ*	2290	This study	pCar2Δ_HygR^{FRT} (pUMa3287)	*car2*	AB33
AB33car2Δ/ AacrtB^{Myc}_NatR	*car2Δ/AacrtB*	2612	This study	pAacrtB^{Myc}_NatR^{FRTm7} (pUMa3707)	*car2*	AB33car2Δ_HygR
AB33car2Δ/AacrtB^{Myc}_NatR/ car1Δ_HygR	*car2Δ/AacrtB/ car1Δ*	3246	This study	pCar1Δ_HygR^{FRT} (pUMa3286)	*car1*	AB33car2Δ/ AacrtB^{Myc}_NatR
AB33car2Δ/AacrtB^{Myc}_NatR/ aat1^{HA}_CbxR	*car2Δ/AacrtB/ aat1*	2706	This study	pAat1^{HA}_CbxR (pUMa3379)	*ip^{s}*	AB33car2Δ/ AacrtB^{Myc}_NatR
AB33car2Δ/AacrtB^{Myc}_NatR/ hmg1$^{NΔ1\text{-}932HA}$_CbxR	*car2Δ/AacrtB/ hmg1$^{NΔ1\text{-}932}$*	2707	This study	pHmg1$^{NΔ1\text{-}932}$_CbxR (pUMa3381)	*ip^{s}*	AB33car2Δ/ AacrtB^{Myc}_NatR
AB33car2Δ/AacrtB^{Myc}_NatR/ idi1^{HA}_CbxR	*car2Δ/AacrtB/ idi1*	2708	This study	pIdi1^{HA}_CbxR (pUMa3382)	*ip^{s}*	AB33car2Δ/ AacrtB^{Myc}_NatR
AB33car2Δ/AacrtB^{Myc}_NatR/ aat1G_CbxR	*car2Δ/AacrtB/ aat1G*	3065	This study	pAat1G_CbxR (pUMa4230)	*ip^{s}*	AB33car2Δ/ AacrtB^{Myc}_NatR
AB33car2Δ/AacrtB^{Myc}_NatR/ hmg1$^{NΔ1\text{-}932}$G_CbxR	*car2Δ/AacrtB/ hmg1$^{NΔ1\text{-}932}$*	3066	This study	pHmg1$^{NΔ1\text{-}932}$G_CbxR (pUMa4231)	*ip^{s}*	AB33car2Δ/ AacrtB^{Myc}_NatR
AB33car2Δ/AacrtB^{Myc}_NatR/ idi1G_CbxR	*car2Δ/AacrtB/ idi1G*	3067	This study	pIdi1G_CbxR (pUMa4232)	*ip^{s}*	AB33car2Δ/ AacrtB^{Myc}_NatR
AB33egfp_CbxR	*egfp*	2229	Köpke, 2011	peGfp_CbxR (pUMa1139)	*ip^{s}*	AB33
AB33upa9mC_HygR/ egfp-skl_CbxR	*upa9mC/egfp-skl*	2386	This study	pUpa9mC_HygR (pUMa2722)	*upa9*	AB33egfp-skl_CbxR
AB33pab1mC_HygR/ upa9G_NatR	*pab1mC/upa9G*	1951	This study	pUpa9G_NatR (pUMa2938)	*upa9*	AB33pab1mC
AB33car2Δ/AacrtB^{Myc}	*car2Δ/AacrtB*	2775	This study	pFLPexpC (pUMa1446)	*car2*	AB33car2Δ/ AacrtB^{Myc}_NatR
AB33car2Δ/AacrtB^{Myc}/ cco1Δ_HygR	*car2Δ/AacrtB/ cco1Δ*	2785	This study	pCco1Δ_HygR (pUMa3281)	*cco1*	AB33car2Δ/ AacrtB^{Myc}
AB33car2Δ/AacrtB^{Myc}/ cco1Δ/idi1^{HA}_NatR	*car2Δ/AacrtB/ cco1Δ/idi1*	3025	This study	pIdi1^{HA}_NatR^{FRTm1} (pUMa4082)	*cco1*	AB33car2Δ/AacrtB^{Myc}/ cco1Δ_HygR
AB33car2Δ/AacrtB^{Myc}/ cco1Δ/idi1^{HA}	*car2Δ/AacrtB/ cco1Δ/idi1*	3077	This study	pFLPexpC (pUMa1446)	*cco1*	AB33car2Δ/AacrtB^{Myc}/ cco1Δ/idi1^{HA}_NatR
AB33car2Δ/AacrtB^{Myc}/ cco1Δ/idi1^{HA}/upp3Δ_HygR	*car2Δ/AacrtB/ cco1Δ/idi1/upp3Δ*	3131	This study	pUpp3Δ_HygR^{FRTm3} (pUMa1556)	*upp3*	AB33car2Δ/AacrtB^{Myc}/ cco1Δ/idi1^{HA}
AB33upp3Δ/egfp_NatR	*upp3Δ/egfp*	2179	This study	peGfp_NatR (pUMa3132)	*upp3*	AB33upp3Δ_HygR
AB33car2Δ/AacrtB^{Myc}/ upp3Δ_HygR	*car2Δ/AacrtB/ upp3Δ*	2884	This study	pUpp3Δ_HygR^{FRTm3} (pUMa1556)	*upp3*	AB33car2Δ/AacrtB^{Myc}
AB33car2Δ/AacrtB^{Myc}/ upp3Δ/CnVS_NatR	*car2Δ/AacrtB/ upp3Δ/CnVS*	3194	This study	pCnVS_NatR^{FRTm2} (pUMa4500)	*upp3*	AB33car2Δ/AacrtB^{Myc}/ upp3Δ_HygR
AB33car2Δ/AacrtB^{Myc}/ upp3Δ/CnVSG_NatR	*car2Δ/AacrtB/ upp3Δ/CnVSG*	3104	This study	pCnVSG_NatR^{FRTm2} (pUMa4356)	*upp3*	AB33car2Δ/AacrtB^{Myc}/ upp3Δ_HygR
AB33car2Δ/AacrtB^{Myc}/ upp3Δ/CnVSG^{NES}_NatR	*car2Δ/AacrtB/ upp3Δ/CnVSG^{NES}*	3192	This study	pCnVSG^{NES}_NatR^{FRTm2} (pUMa4499)	*upp3*	AB33car2Δ/AacrtB^{Myc}/ upp3Δ_HygR
AB33car2Δ/AacrtB^{Myc}/ cco1Δ/idi1^{HA}/ upp3Δ/CnVSG^{NES}_NatR	*car2Δ/AacrtB/ cco1Δ/idi1/ upp3Δ/CnVSG^{NES}*	3195	This study	pCnVSG^{NES}_NatR^{FRTm2} (pUMa4499)	*upp3*	AB33car2Δ/AacrtB^{Myc}/ cco1Δ/idi1^{HA}/ upp3Δ_HygR
AB33car2Δ/AacrtB^{Myc}/ upp3Δ/cop6_NatR	*car2Δ/AacrtB/ upp3Δ/cop6*	3193	This study	pCop6_NatR^{FRTm2} (pUMa4496)	*upp3*	AB33car2Δ/AacrtB^{Myc}/ upp3Δ_HygR
AB33car2Δ/AacrtB^{Myc}/ upp3Δ/cop6G_NatR	*car2Δ/AacrtB/ upp3Δ/cop6G*	2944	This study	pCop6G_NatR^{FRTm2} (pUMa4089)	*upp3*	AB33car2Δ/AacrtB^{Myc}/ upp3Δ_HygR
AB33car2Δ/AacrtB^{Myc}/ upp3Δ/cop6G^{NES}_NatR	*car2Δ/AacrtB/ upp3Δ/cop6G^{NES}*	3078	This study	pCop6G^{NES}_NatR^{FRTm2} (pUMa4316)	*upp3*	AB33car2Δ/AacrtB^{Myc}/ upp3Δ_HygR
AB33car2Δ/AacrtB^{Myc}/ cco1Δ/idi1^{HA}/ upp3Δ/cop6G^{NES}_NatR	*car2Δ/AacrtB/ cco1Δ/idi1/ upp3Δ/cop6G^{NES}*	3148	This study	pCop6G^{NES}_NatR^{FRTm2} (pUMa4316)	*upp3*	AB33car2Δ/AacrtB^{Myc}/ cco1Δ/idi1^{HA}/ upp3Δ_HygR

Supplementary Table S3: Description of plasmids used for *U. maydis* strains generation

Plasmid name	pUMa	Resistance cassette	Short description
pCco1Δ_HygR	3281	*Sfi*I-insert MF1hs	Plasmid for generating deletion mutants of *cco1*. The hygromycin B resistance cassette contains FRT sites (GAAGTTCCTATTCTCTAGAAA GTATAGGAACTTC) at both ends for recycling by Flp recombinase. The cassette is flanked by the regions, 1.1 kb upstream and 0.8 kb downstream of *cco1*. The flanking regions were amplified by PCR using oMB981/oMB982 and oMB983/oMB984 and UM521 wild type DNA as template.
pCar1Δ_HygR	3286	*Sfi*I-insert MF1hs	Plasmid for generating deletion mutants of *car1*. The hygromycin B resistance cassette contains FRT sites at both ends for recycling by Flp recombinase. The cassette is flanked by the regions, 0.9 kb upstream and 0.6 kb downstream of *car1*. The flanking regions were amplified by PCR using oUP134/oMB998 and oMB999/oUP009 and UM521 wild type DNA as template.
pCar2Δ_HygR	3287	*Sfi*I-insert MF1hs	Plasmid for generating deletion mutants of *car2*. The hygromycin B resistance cassette contains FRT sites at both ends for recycling by Flp recombinase. The cassette is flanked by the regions, 0.9 kb upstream and 0.9 kb downstream of *car2*. The flanking regions were amplified by PCR using oUP014/oUP015 and oUP016/oUP017 and UM521 wild type DNA as template.
pUpp3Δ_HygR	1556	*Sfi*I-insert MF1hs	Plasmid for generating deletion mutants of *upp3*. The hygromycin B resistance cassette contains FRTm3 sites (GAAGTTCCTATTCTCCAGA AAGTATAGGAACTTC) at both ends for recycling by Flp recombinase. The cassette is flanked by the regions, 1.5 kb upstream and 1.9 kb downstream of *upp3*. The flanking regions were amplified by PCR using UM521 wild type as template. Published (Sarkari, 2014).
pFLPexpC	1446	CbxR	Plasmid for producing Flp recombinase, which is under the control of P_{crg1} promoter and the gene expression is induced in the presence of arabinose. Containing the carboxin resistance cassette. Published (Khrunyk, 2010)
peGfp_NatR	3132	*Sfi*I-insert of pMF5-1n	Plasmid for producing eGfp with the nourseothricin resistance cassette at the *upp3* locus. The expression of *egfp* is under the control of P_{otef} and T_{nos} terminator.
pAacrtB^{Myc}_NatR^{FRTm7}	3707	*Sfi*I-insert of pMF5-1n	Plasmid for producing codon-optimized *Aa*CrtB, which is C-terminally fused to 3× Myc tag with the nourseothricin resistance cassette containing FRTm7 (GAAGTTCCTATTCTCTATAAAGTATAGGAACTTC), at the *car2* locus. The expression of *AacrtB*^{Myc} is under the control of P_{rpl40} (1 kb upstream of the ORF) promoter and T_{nos} terminator.
pAat1^{HA}_CbxR	3379	CbxR for integration at the *ip*^{s} locus	Plasmid for producing Aat1 N-terminally fused to 3× HA tag at the *ip*^{s} locus. The gene expression is under the control of P_{otef} promoter and T_{nos} terminator.
pHmg1$^{NΔ1\text{-}932HA}$_CbxR	3381	CbxR for integration at the *ip*^{s} locus	Plasmid for producting N-terminally truncated from aa 1 to 932 of Hmg1, which is N-terminally fused to 3× HA tag, at the *ip*^{s} locus. The gene expression is under the control of P_{otef} promoter and T_{nos} terminator.
pIdi1^{HA}_CbxR	3382	CbxR for integration at the *ip*^{s} locus	Plasmid for producing Idi1 N-terminally fused to 3× HA tag at the *ip*^{s} locus. The gene expression is under the control of P_{otef} promoter and T_{nos} terminator.
pAat1G_CbxR	4230	CbxR for integration at the *ip*^{s} locus	Plasmid for producing Aat1 N-terminally fused to eGfp at the *ip*^{s} locus. The gene expression is under the control of P_{tef} promoter and T_{nos} terminator.
pHmg1$^{NΔ1\text{-}932}$G_CbxR	4231	CbxR for integration at the *ip*^{s} locus	Plasmid for producing N-terminally truncated from aa 1 to 932 of Hmg1, which is N-terminally fused to eGfp, at the *ip*^{s} locus. The gene expression is under the control of P_{tef} promoter and T_{nos} terminator.
pIdi1G_CbxR	4232	CbxR for integration at the *ip*^{s} locus	Plasmid for producing Idi1 N-terminally fused to eGfp at the *ip*^{s} locus. The gene expression is under the control of P_{tef} promoter and T_{nos} terminator.
pUpa9mC_HygR	2722	*Sfi*I-insert MF1hs	Plasmid for producing Upa9 C-terminally fused to mCherry with the hygromycin B resistance cassette at the *upa9* locus. The insert construct is flanked by the regions, 1.1 kb of *upa9* (UMAG_06200) ORF and 1.0 kb downstream of *upa9*. The flanking regions were amplified by PCR using oDD527/oDD605 and oDD634/oDD635 and UM521 wild type DNA as template. The gene expression is under the control of the native promoter and T_{nos} terminator.
peGfp-skl_CbxR	3141	CbxR for integration at the *ip*^{s} locus	Plasmid for producing eGfp C-terminally fused to SKL at the *ip*^{s} locus. The gene expression is under the control of P_{otef} promoter and T_{nos} terminator.
pUpa9G_NatR	2938	*Sfi*I-insert of pMF5-1n	Plasmid for producing Upa9 C-terminally fused to eGfp with the nourseothricin resistance cassette at the *upa9* locus. The insert construct is flanked by the regions, 1.1 kb of *upa9* ORF and 1.0 kb downstream of *upa9*. The flanking regions were amplified by PCR using oDD527/oDD605 and oDD634/oDD635 and UM521 wild type DNA as

			template. The gene expression is under the control the native promoter and T_{nos} terminator.
pIdi1^{HA}_NatR^{FRTm1}	4082	*Sfi*I-insert of pMF5-1n	Plasmid for producing Idi1 N-terminally fused to 3× HA tag with the nourseothricin resistance cassette containing FRTm1 sites (GAAGTTCC TATTCTCGAGAAAGTATAGGAACTTC) at both ends for recycling by Flp recombinase at the *cco1* locus. The gene expression is under the control of P_{rpl10} (1 kb upstream of the ORF) promoter and T_{hsp70} terminator.
pCnVS_NatR^{FRTm2}	4500	*Sfi*I-insert of pMF5-1n	Plasmid for producing dicodon-optimized CnVS with the nourseothricin resistance cassette containing FRTm2 sites (GAAGTTCCTATTCTCAAG AAAGTATAGGAACTTC) at both ends for recycling by Flp recombinase at the *upp3* locus. The gene expression is under the control of P_{otef} promoter and T_{nos} terminator.
pCnVSG_NatR^{FRTm2}	4356	*Sfi*I-insert of pMF5-1n	Plasmid for producingexpressing dicodon-optimized CnVS, which is N-terminally fused to eGfp with the nourseothricin resistance cassette containing FRTm2 sites at both ends for recycling by Flp recombinase, at the *upp3* locus. The gene expression is under the control of P_{otef} promoter and T_{nos} terminator.
pCnVSG^{NES}_NatR^{FRTm2}	4499	*Sfi*I-insert of pMF5-1n	Plasmid for producing dicodon-optimized CnVS, which is N-terminally fused to eGfp^{NES} with the nourseothricin resistance cassette containing FRTm2 sites at both ends for recycling by Flp recombinase, at the *upp3* locus. The gene expression is under the control of P_{otef} promoter and T_{nos} terminator.
pCop6_NatR^{FRTm2}	4496	*Sfi*I-insert of pMF5-1n	Plasmid for producing dicodon-optimized Cop6 with the nourseothricin resistance cassette containing FRTm2 sites at both ends for recycling by Flp recombinase, at the *upp3* locus. The gene expression is under the control of P_{otef} promoter and T_{nos} terminator.
pCop6G_NatR^{FRTm2}	4089	*Sfi*I-insert of pMF5-1n	Plasmid for producing dicodon-optimized Cop6, which is N-terminally fused to eGfp with the nourseothricin resistance cassette containing FRTm2 sites at both ends for recycling by Flp recombinase, at the *upp3* locus. The gene expression is under the control of P_{otef} promoter and T_{nos} terminator.
pCop6G^{NES}_NatR^{FRTm2}	4316	*Sfi*I-insert of pMF5-1n	Plasmid for producing dicodon-optimized Cop6, which is N-terminally fused to eGfp^{NES} with the nourseothricin resistance cassette containing FRTm2 sites at both ends for recycling by Flp recombinase, at the *upp3* locus. The gene expression is under the control of P_{otef} promoter and T_{nos} terminator.

Supplementary Table S4: DNA oligonucleotides used in this study

Designation	Nucleotide sequence (5'→ 3')	Remarks
oMB980	GCAGTCTTGGCGAGCTATTC	*cco1* U1
oMB981	GGTCTCGCCTGCAATATTTCACCATTATTCTCTTCATTTACTG	*cco1* U2
oMB982	GGTCTCCAGGCCCGGGCAAATGCCTATCGAG	*cco1* U3
oMB983	GGTCTCCGGCCGCGAGATGGAAGTGCCTCGC	*cco1* D1
oMB984	GGTCTCGCTGCAATATTTCTTGCTAGGACTGAAAGCG	*cco1* D2
oMB985	GACAATGGTGCTTTGCAGGG	*cco1* D3
oMB986	CTAGGTCTCGTGTCTGAACTCGCACCCAAAGTTG	*cco1* UF_fw
oMB987	CTAGGTCTCAGACACCCTCCGAATGACTATTTGTAC	*cco1* UF_rv
oUP054	CCGCGATTCAGTCAGGTCAG	*cco1* P1
oUP055	GAATCTATCAGTGACGGCAC	*cco1* P2
oMB996	GCATCTGTCGCAGCAACATC	*car1* U1
oUP134	GGTCTCGCCTGCAATATTGATGAATAGCCTCTTTGCCG	*car1* U2
oMB998	CTAGGTCTCCAGGCCCTTGACCAAGACAAGAACCTCCG	*car1* U3
oMB999	CTAGGTCTCCGGCCGGAGCGTCTACACAACCGGG	*car1* D1
oUP009	CTAGGTCTCGCTGCAATAATTTCGACTTCCATACAATGCTCGC	*car1* D2
oUP010	AAGCTGCTGATGCCGCCTTG	*car1* D3
oUP011	CTAGGTCTCGTGTCGAGGCGAGGTATAAGCAATG	*car1* DF_fw
oUP012	CTAGGTCTCAGACACCAAGTTGACGTTCTTGTCTC	*car1* DF_rv
oUP013	TCTCCCTGCATGGTAGTAGC	*car2* U1
oUP014	GGTCTCGCCTGCAATAATACAATAATAAATCGAAGCGGTTCAC	*car2* U2
oUP015	GGTCTCCAGGCCGACTGGCACTGATTGGTCAAC	*car2* U3
oUP016	GGTCTCCGGCCAGCATGCACACATGCATCATC	*car2* D1
oUP017	GGTCTCGCTGCAATATTCACTGCGAAGCGGAGGATC	*car2* D2
oUP018	CGAAACACAGAAGCGATGAG	*car2* D3
oUP056	GACAACGCCATGGGACAATC	*car2* P1
oUP057	CATAGACGGCGTCGACAATG	*car2* P2
oUP186	CTAGGCGCGCCCCAGCTTTCTACCAAACCGCC	*aat1*_fw
oUP187	CTACCGCGGTTAGTTCTCACGCTTGATAATGATAG	*aat1*_rv
oUP266	CTAGGCGCGCCCTGCAGGATGCTACCTATGTTAC	*hmg1*$^{N\Delta 1\text{-}932}$_fw
oUP267	CTACCGCGGCTATGTGAGAGAAGATGCTCGAC	*hmg1*$^{N\Delta 1\text{-}932}$_rv
oUP268	CTAGGCGCGCCTCGACCGCCACCGTCACCGAG	*idi1*_fw
oUP269	CTACCGCGGTCAGAGGAGGCGGTGAATGC	*idi1*_rv
oDD527	GGTCTCGCCTGCAATATTGCCAATGGCACTAC	*Upa9* U2
oDD605	GGTCTCGTGGCCAAAGAAGACCAGGCAGCATAG	*Upa9* U3
oDD634	GGTCTCCGGCCTAGTAGGGTCCAACGTCTAC	*Upa9* D1
oDD635	GGTCTCCCTGCCAATATTCGACCTCGAGGCCGAG	*Upa9* D2
oMB935	GATCCCATGGTGAGCAAGGGCGAGGAG	SKL_fw
oMB936	GCGGCCGCTTTAGAGCTTGGACTTGTACAGCTCGTCCAT	SKL_rv
oUM121	CTACCTGCAGGGGTACTGATGACGATGACGAAGAAG	P*rps1*_fw
oUM122	CTACCATGGGATGAATCGATATGTCTTGAGGAAG	P*rps1*_rv
oUM123	CTACCTGCAGGGTATGTGTAGAGGTGGCCTTG	P*rpl10*_fw
oUM124	CTACCATGGCTTGAATACTGTTGGATGGGAGG	P*rpl10*_rv
oAB049	CATGACCAAGAAGTTTGGCACGCTCACCATCT	NES_fw
oAB050	CCGGAGATGGTGAGCGTGCCAAACTTCTTGGT	NES_rv

Supplementary Table S5: Codon optimized DNA sequences for expression in *U. maydis*

**Phytoene synthase *Aa*CrtB from *Agrobacterium aurantiacum***
ATGTCGGACCTGGTTCTTACGTCTACCGAAGCTATTACACAAGGCTCGCAGTCGTTCGCTACAGCGGCGAAGCTTATGCCT<br>CCTGGCATCCGCGACGATACAGTCATGCTCTATGCGTGGTGCCGTCATGCTGATGACGTGATTGATGGTCAGGCGCTCGGA<br>AGCCGTCCCGAGGCGGTGAACGACCCACAAGCTCGCCTTGATGGACTCCGCGCAGACACCCTGGCCGCCCTTCAGGGTGA<br>TGGTCCGGTGACCCCTCCATTTGCTGCATTGCGAGCCGTGGCACGACGACACGATTTTCCTCAAGCGTGGCCTATGGATCT<br>CATTGAGGGTTTCGCAATGGATGTTGAGGCTCGTGATTACCGTACACTCGACGACGTGCTGGAGTACAGCTATCATGTTGC<br>CGGAATTGTCGGCGTCATGATGGCCCGAGTCATGGGTGTCCGCGATGATCCTGTGTTGGACCGAGCCTGTGACCTGGGTCT<br>GGCATTCCAGCTGACAAATATCGCACGCGACGTCATCGACGACGCTCGAATTGGACGATGCTACCTTCCAGGAGACTGGC<br>TGGACCAAGCGGGCGCACGAGTGGACGGACCAGTTCCAAGCCCAGAGCTTTACACCGTCATTTTGCGTCTGTTGGACGCA<br>GCCGAATTGTATTACGCCTCTGCACGAGTTGGCTTGGCAGATTTGCCTCCGCGATGCGCGTGGAGCATCGCCGCTGCACTC<br>CGCATCTATCGTGCCATTGGACTGCGTATCCGCAAAGGAGGACCAGAGGCATACCGTCAACGTATTTCCACTTCCAAAGCT<br>GCTAAAATTGGACTCCTGGGCATTGGCGGCTGGGACGTTGCACGCAGCCGCCTCCCCGGCGCGGGTGTCAGCCGCCAAGG<br>CCTGTGGACTCGACCGCACCATGCATAG
**(+)-valencene synthase CnVS from *Callitropsis nootkatensis***
ATGGCCGAGATGTTCAACGGCAACTCGAGCAACGACGGCTCGTCGTGTATGCCCGTCAAGGACGCGCTGCGCCGCACCGG<br>CAACCACCACCCCAACCTCTGGACCGACGACTTTATCCAGTCGCTCAACTCGCCCTACTCGGACTCGTCGTACCACAAGCA<br>CCGCGAGATCCTCATCGACGAGATCCGCGACATGTTCTCCAACGGCGAGGGCGACGAGTTCGGTGTGCTCGAGAACATCT<br>GGTTCGTCGACGTCGTCCAGCGTCTCGGCATCGACCGCCACTTCCAGGAGGAGATCAAGACGGCGCTCGACTACATCTACA<br>AGTTCTGGAACCACGATTCGATCTTTGGCGACCTCAACATGGTCGCTCTCGGTTTCCGCATCCTGCGTCTCAACCGCTACGT<br>CGCTTCGAGCGACGTCTTCAAGAAGTTCAAGGGCGAGGAGGGTCAGTTCTCGGGCTTCGAGTCGTCCGACCAGGACGCCA<br>AGCTCGAAATGATGCTCAACCTGTACAAGGCTTCCGAGCTCGACTTCCCCGACGAGGACATTCTCAAGGAGGCGCGTGCTT<br>TTGCTTCGATGTACCTCAAGCACGTCATCAAGGAGTACGGTGACATCCAGGAGAGCAAGAACCCGCTGCTCATGGAGATC<br>GAGTACACCTTCAAGTACCCTTGGCGTTGCCGTCTGCCCCGTCTCGAGGCGTGGAACTTTATCCACATCATGCGTCAGCAG<br>GACTGCAACATCTCGCTCGCCAACAACCTCTACAAGATCCCCAAGATCTACATGAAAAAGATCCTCGAGCTCGCCATCCTC<br>GACTTCAACATCCTCCAGTCGCAGCACCAGCACGAGATGAAGCTCATCTCGACCTGGTGGAAGAACTCGTCGGCCATCCA<br>GCTCGACTTTTTCCGTCACCGCCACATCGAGTCGTACTTCTGGTGGGCCTCGCCCCTCTTCGAGCCCGAGTTCTCGACGTGC<br>CGCATCAACTGCACCAAGCTCTCGACCAAGATGTTCCTGCTCGACGACATCTACGACACCTACGGCACCGTCGAAGAGCTC<br>AAGCCCTTCACCACCACGCTCACCCGCTGGGACGTCTCGACCGTCGACAACCACCCCGACTACATGAAGATCGCCTTCAAC<br>TTTAGCTACGAGATCTACAAGGAGATCGCCTCGGAAGCTGAGCGCAAGCACGGTCCTTTTGTCTACAAGTACCTCCAGTCG<br>TGCTGGAAGTCGTACATCGAGGCGTACATGCAGGAGGCCGAGTGGATCGCCAGCAACCACATCCCTGGTTTCGACGAGTA<br>CCTCATGAACGGTGTCAAGTCGTCGGGTATGCGCATCCTCATGATTCACGCGCTCATCCTCATGGACACGCCGCTCTCCGA<br>CGAGATTCTCGAACAGCTCGACATCCCTTCGTCGAAGTCGCAGGCTCTGCTCTCGCTCATCACGCGTCTCGTCGACGACGT<br>CAAGGACTTTGAAGACGAGCAGGCGCATGGCGAGATGGCTTCGTCGATCGAGTGCTACATGAAGGACAACCACGGTTCGA<br>CGCGCGAGGATGCGCTCAACTACCTCAAGATCCGCATCGAGTCGTGCGTCCAGGAGCTCAACAAGGAGTTGCTCGAGCCC<br>TCGAACATGCACGGCTCGTTCCGCAACCTCTACCTCAACGTCGGCATGCGTGTCATCTTCTTCATGCTCAACGACGGTGAC<br>CTCTTCACCCACTCGAACCGCAAGGAGATCCAGGACGCCATCACCAAGTTCTTTGTCGAGCCCATCATCCCCTGA
**The fungal sesquiterpenoid synthase Cop6 from *Coprinopsis cinerea***
ATGCCTGCTGCTCTGCCCTACAACGTCTCGCGCGACAACAAGTGGGATATCAAGAAGATCATCCAGGACTTTTTCAAGCGC<br>TGCGATGTGCCCTACCAGGTCATCCCCTACGACACCGAGCTCTGGAACGCCTGCCTCAAGCGTGCCAAGGAGAAGGGCTA<br>CCCCGTCGAGCCCGACTCGCCCATGTCGCTCTACCGCAGCTTCAAGGTCGGTGTCGTCATCACGCGCACCTCGTACGGTCA<br>CATCCAGGACTACGAGATCCTCATCTGGGTCGCCACCTTCACCGCCTTTGTCACCTACGCCGACGACGCCTTCCAGGAGGA<br>CATCCAGCACCTCCACAGCTTCGCTCGCACCTTCCTCCAGAACGAAAAGCACGAGCATCCCGTCCTCGAGGCCTTTGCCCA<br>GTTCCTGCGCGAGTCGTCGATCCGATTCTCGCACTTTGTCGCCAACACCGTCGTCTCGTCGGCGCTGCGCTTCATGATGTCG<br>ATCGCGCTCGAGTTCGAGGGTCAGAACGTCTCGGTCTCGACCGAGGCGCGTGAGTACCCTGGCTACATCCGCATCCTCTCG<br>GGTCTCTCGGACATCTACGCGCTCTTCGCCTTCCCCATGGACCTGCCGCGTTCCACCTACATCCAGGCCTTCCCCGAGCAGA<br>TCGACTACATCAACGGCACCAACGACCTGCTCTCGTTCTACAAGGAGGAGCTCGACTGCGAGACCGTCAACTTTATCTCGG<br>CCGCTGCCACCTCGCAGCAGGTCAGCAAGCTCGAGGTGCTGCGCAACGCCGCCGAGAAGGCCGCCTACTCGTACGACGTC<br>GTCGTCAACGTGCTCAAGCCCTACCCCGAGGCGCTTGCCGCCTGGAAGTCGTTTGCTCGCGGCTTCTGCTACTTCCACACCT<br>CGTCGCCACGCTACCGTCTCGGCGAGATGTTCCACGACTTTGAGCACGACCTCGTCTGCAAGTGCGCCTCGTGCACCGAGA<br>TCTGA
